# Diabetic Retinopathy Detection: AI Models and Approaches

**DOI:** 10.1155/joph/8857887

**Published:** 2026-04-26

**Authors:** Meftah Mohamed Mohamed Madi, Peter Clarke- Farr, Dirk Bester

**Affiliations:** ^1^ Department of Biomedical Sciences, Faculty of Health and Wellness Sciences, Cape Peninsula University of Technology, Cape Town, South Africa, cput.ac.za; ^2^ Department of Ophthalmic Sciences, Faculty of Health and Wellness Sciences, Cape Peninsula University of Technology, Cape Town, South Africa, cput.ac.za; ^3^ Department of Biomedical Sciences, Cape Peninsula University of Technology, Symphony Way, Bellville, Cape Town, P.O. Box 1906, Bellville 7535, South Africa, cput.ac.za

## Abstract

**Background:**

Diabetic retinopathy (DR), a major cause of vision loss worldwide, results from chronic diabetes damage to retinal blood vessels. Vision loss can be prevented if DR is detected early, but traditional retinal screening by eye care takes time and expertise. Recent advances in AI technology, including classical machine learning and deep learning, can be more accurate in DR detection. This article provides a comprehensive review of current AI models and approaches of DR screening.

**Methods:**

We searched PubMed, Web of Science, Scopus, ScienceDirect, and EBSCOhost using the keywords: diabetes, retinopathy, screening, and early detection. The search was limited to English language and studies published between 2020 and 2025.

**Results:**

The findings suggest that AI models have become crucial for early DR diagnosis. While traditional machine learning previously lacked effectiveness, deep learning has now significantly improved diagnostic performance. The models, such as the URNet system, the vision transformer (ViT) model, the ResNet‐50 and EfficientNetB0 models, the DenseNet model, and the ResNet‐18 model, have achieved high‐performance metrics using publicly available datasets. DR screening devices, like ADX‐DR, have shown commendable performance. The EyeArt modality demonstrated exceptional sensitivity across diverse populations, detecting around 98.5% of vision‐threatening DR, while Google AI matched specialist performance in specificity and surpassed it in sensitivity.

**Conclusion:**

AI methods using deep learning frameworks such as CNNs have attained expert‐level accuracy in DR classification, in addition to real‐world validation. Semiautonomous systems like the IDx‐DR and EyeArt have robust clinical performance and scalability, especially in countries with few ophthalmologists. Although research has been mainly conducted in Asia, there is a lack of research from Africa and low‐income countries. Future techniques, including ensemble models and federated learning, will enhance accuracy and reliability further, aiding early diagnosis and prevention of vision loss globally.

## 1. Introduction

Diabetes is a fast‐growing chronic disease that presents a major threat to individual health as the global population continues to expand. This rise is primarily due to busy and stressful lifestyles, poor dietary choices, and limited awareness of the condition. Diabetes has several effects on the body, but diabetic retinopathy (DR) is one of the most serious side effects. DR is a leading cause of visual impairment worldwide, resulting from long‐term diabetes damaging the retinal blood vessels often following persistent high blood sugar levels [1]. Early detection and regular screening with timely treatment of DR can prevent visual loss. However, if left untreated, DR can cause severe vision loss or even blindness. Hence, models for the early detection and diagnosis of DR are crucial [2]. Approximately 37 million people worldwide are blind as a result of DR [3]. The primary goal of current DR therapies is therefore to decrease or stop further vision loss by limiting proliferative retinopathy, highlighting the importance of routine eye screening. Previously, the retinal fundus photos obtained during the screening process took significant time to be assessed manually and also relied on experienced eye care professionals [1]. Over recent years, artificial intelligence (AI)–based solutions (such as traditional machine learning [ML] and deep learning [DL]) have been widely implemented to automate and enhance early DR detection. These AI models process the retinal images and forecast the type of DR and its severity [4]. The majority of these models work similarly but may differ in effectiveness and accuracy of their findings [5]. Despite the established role of AI in the screening and diagnosis of DR, empirical evidence of the challenges, strengths, and weaknesses of the existing models is restricted to individual research papers. Since this is a rapidly evolving field, and the systematic reporting of current AI models that have been adopted for the detection of DR, especially over the last 5 years is limited, this paper intends to provide a comprehensive analysis of the latest literature published on this topic.

## 2. Methodology

This article aims to provide a comprehensive review of current AI models used for the detection of DR based on published peer‐reviewed scientific articles. While this paper is intended to serve as a review article, the authors adopted the PRISMA protocol for the reporting of systematic reviews as a means to ensure quality and scientific rigor of the research process.

### 2.1. Search Strategy

The search strategy was comprehensive and sought scientific databases including EBSCOhost, Web of Science, PubMed, Scopus, and ScienceDirect. The keywords searched included diabetes AND retinopathy AND screening AND early AND detection. The search was limited to full‐text peer‐reviewed scientific articles, English, and publication studies between 2020 and 2025. The search strategy is presented and criteria in Tables 1 and 2.

**TABLE 1:** Search strategy.

Name of database	Search terms	Number of articles found
EBSCOhost	Academic Search Premier, Art & Architecture Complete, Business Source Premier, CINAHL with Full Text, eBook Collection (EBSCOhost), ERIC, GreenFILE, Health Source ‐ Consumer Edition, Health Source: Nursing/Academic Edition, Library, Information Science & Technology Abstracts, MasterFILE Premier, MasterFILE Premier Reference eBook Subscription (EBSCOhost), MEDLINE, Newspaper Source, Regional Business News, SPORTDiscus with Full Text, Teacher Reference Center Diabetic AND retinopathy AND model AND screening AND early AND detection (All fields). Limited to Academic Journal, full text, English (languages), and Publication year within last 5 years	109

Web of Science	Diabetic AND retinopathy AND model AND screening AND early AND detection (All fields) and Article (document type) and English (Languages) and (Open Access) and 2020 to 2025 (Publication Years)	115

PubMed	Diabetic[Title/Abstract] AND retinopathy[Title/Abstract] AND model[Title/Abstract] AND screening[Title/Abstract] AND early[Title/Abstract] AND detection[Title/Abstract] filters applied: Free full text, English language and Publication year within last 5 years	73

Scopus	TITLE‐ABS‐KEY (Diabetic AND retinopathy AND model AND screening AND early AND detection) AND PUBYEAR > 2019 AND PUBYEAR < 2026 AND PUBYEAR > 2019 AND PUBYEAR < 2026 AND PUBYEAR > 2019 AND PUBYEAR < 2026 AND (LIMIT‐TO (SRCTYPE, “j”)) AND (LIMIT‐TO (OA, “all”)) AND (LIMIT‐TO (LANGUAGE, “English”)) AND (LIMIT‐TO (DOCTYPE, “re”) OR LIMIT‐TO (DOCTYPE, “ar”)).	122

ScienceDirect	diabetic AND retinopathy AND model AND screening AND early AND detection.(Title, abstract or author‐specified keywords) limited to article type (journal articles) and (review articles), and (Publication years) between 2020 and 2025	44

		Total 463

**TABLE 2:** Search criteria.

#	Database	Language	Peer reviewed	All open access	Core collection	Full text	Free full text	Research articles	Journal article	Review articles	Academic journal	2020–2025
1	EBSCOhost	✓				✓					✓	✓
2	Web of Science	✓		✓	✓			✓				✓
3	PubMed	✓				✓						✓
4	Scopus	✓		✓				✓	✓	✓		✓
5	ScienceDirect	✓						✓		✓		✓

### 2.2. Study Selection

The relevant studies that were identified from a database search were saved into the researcher’s computer and Mendeley reference software for further screening. Duplicated articles from different databases were identified and removed. After title and abstract screening, identified articles were assessed for eligibility criteria; the inclusion and exclusion criteria are provided in Table 3. Articles that were not full‐text articles were identified and excluded. Finally, the eligible articles that deal with the application of AI models for the early diagnosis and screening of DR were included in the review. The included articles were saved in an Excel spreadsheet for the extraction of data, and the characteristics of the included articles are presented in tables and attached in Appendix A (Table A1):.

**TABLE 3:** Criteria for included and excluded articles.

#	Included articles	Excluded articles
1	Articles dealing with diabetic retinopathy screening	Diabetes articles that did not include a specific reference to the screening of diabetic retinopathy
2	Articles focused on AI models used for the early detection of diabetic retinopathy	Articles that did not focus on the AI models used for the early detection of diabetic retinopathy
3	Full‐text article availability	Articles are not in full text
4	Articles conducted between 2020 and 2025	Articles are not written in English

## 3. Results

**FIGURE 1:**
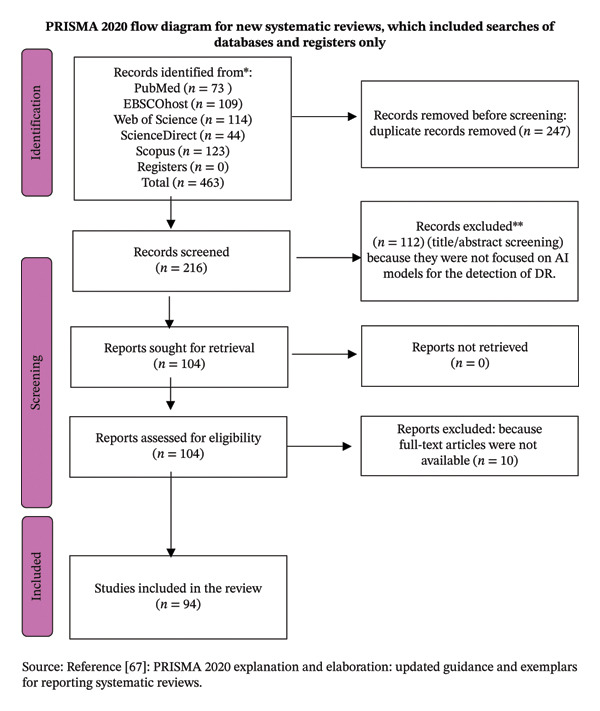
Prisma flow diagram for included and excluded studies.

A total of 463 studies were identified from all databases, and 216 studies were identified after removing duplicate studies. One hundred and twelve studies were excluded from screening title and abstract; the reason for exclusion was that they were not focused on the AI models for the diagnosis and screening of DR. One hundred and four studies were assessed for eligibility, and 10 studies were excluded because they were not available in full text. A total of 94 studies were included in the final review, as presented in Figure 1. The characteristics of the included studies’ tables are reflected in Appendix A (Table A1): 61 included studies with deep learning approaches; Appendix B (Table A2): 13 included studies with a ML approach; and Appendix C (Table A3): 20 included studies with other approaches. These appendix tables provide the article name, date of publication, journal name, and study location (Table 4). The following provides the examples of deep learning models for the screening and grading of DR and reports the model’s name, approach, data source, strengths and weaknesses, and performance metrics.

**TABLE 4:** Examples of deep learning models for diabetic retinopathy.

Article name and citation	Name of the model	Model approach	Data source (images)	Performance metrics
URNet system for recommending referrals for the community screening of diabetic retinopathy based on deep learning [25]	The URNet system utilized ResNet‐50 as a pretrained model	Deep learning methods	The DDR dataset (it contains 13,673 fundus images)	Achieving 94.4% accuracy and 94.5% precisions, and 96.2% recall and 95% F1 score
Research on grading detection methods for diabetic retinopathy based on deep learning [22]	The vision transformer (ViT) model	Deep learning approach	The EyePACS dataset was used for model training (consists of 35,126 retinal images of diverse resolutions and contrasts)	The model achieved an accuracy of 89.35% across datasets. Precision was reported at 90.12%, indicating strong performance. Detection accuracy reached around 0.88 in validation
Multiclass adaptive boosting approach for diabetic retinopathy prediction using diabetic retinal images [89]	VGG16 pretrained model	A novel multiclass adaptive boosting approach	Kaggle repository dataset	The model achieved an accuracy rate of 91.6% with a kappa score of 0.883
MCBM: Implementation of multiclass and transfer learning algorithm based on a deep learning model for early detection of diabetic retinopathy [7]	EfficientNet model + ResNet‐50	The proposed model implemented five deep learning models based on CNN, EfficientNet, VGG 16, ResNet‐50, and MobileNet	Kaggle’s diabetic retinopathy detection (a total of 3662 retinal images were collected)	Efficient Net achieved a training accuracy of 0.9342 and a testing accuracy of 0.8181. RESNET‐50 achieved training accuracy of 0.9329 and testing accuracy of 0.8116. Average Top1 accuracy across models is 77%, and Top5 accuracy is 93%
Fundus image based diabetic retinopathy detection using EfficientNetB3 with squeeze and excitation block [20]	EfficientNetB3 model with squeeze and excitation block	Deep learning approach	APTOS‐2019 dataset (3662 color fundus images)	88.44% accuracy, 98.00% specificity, 15 84.00% precision, 83.00% sensitivity, and 83.00% F1 score
Dual‐branch deep learning network for detection and stage grading of diabetic retinopathy [8]	Deep dual‐branch model	The model utilizes transfer learning for feature extraction. It employs ResNet‐50 and EfficientNetB0 as pretrained models	APTOS 2019 dataset (contains 3662 fundus images for training and testing)	Accuracy of 98.50%, a sensitivity of 99.46%, and a specificity of 97.51%
Development of revised ResNet‐50 for diabetic retinopathy detection [9]	The ResNet‐50 revised model	This study compares the performance of DR grading using the ResNet‐50 revised and other popular CNN models	DR dataset from Kaggle, which includes 35,126 fundus images, of which 25,805 are normal (without disease) and 9321 exhibit diabetic retinopathy (DR)	The revised ResNet‐50 model achieved a train accuracy of 0.8395 and a test accuracy of 0.7432
An efficient DenseNet for diabetic retinopathy screening [14]	Efficient DenseNet	A novel deep learning framework	Asia‐Pacific Tele‐Ophthalmology Society (APTOS) image dataset, containing 5590 retinal images, which was augmented to 13,000 images for the study	The proposed model achieved a classification accuracy of 98.40%. High recall and specificity values were reported for severity grading. The framework demonstrated a precision rate of about 74.58%
A refined ResNet18 architecture with Swish activation function for diabetic retinopathy classification [10]	The ResNet‐18 model	The model utilizes the ResNet‐18 architecture with the Swish activation function	The dataset consists of 5371 fundus images. Images were obtained from the APTOS Kaggle database	93.51% accuracy, 93.42% sensitivity, precision 93.77% precision, and 93.59% F1 score
Ensemble EfficientNet: a novel technique for identification, classification, and prediction of diabetic retinopathy [54]	Ensemble EfficientNet	The model uses deep learning and transfer learning approaches	The images were sourced from the Kaggle DR dataset	The model achieved an accuracy of 95% in D.R. detection Recall rate for the model was reported at 97% Precision was noted to be 0.95, surpassing other models. The AUC value for the model was 0.98, indicating excellent performance
Ensemble of multistage deep convolutional neural networks for automated grading of diabetic retinopathy using image patches [55]	An ensemble of deep CNNs for DR	Deep learning approach	DIARETDB and STARE	The overall classification accuracy achieved is 96.2% with MPDCNN Individual accuracies for classes are 97%, 95%, 96%, and 97% Performance measures include TPR, TNR, PPV, QI, F score, and MCR

### 3.1. Quality Assessment of Included Studies

Risk of bias was evaluated using the QUADAS‐2 tool for diagnostic accuracy tests; QUADAS‐2 was applied to the following four domains to assess the included studies: selection of patients, index test, reference standard, flow, and timing. The risk of bias was as follows, which we judged it as high risk, low risk, or unclear risk depending on the domain [101].

• Domain 1: Patient selection
   Risk of bias
   Low risk: 0 studies (0.0%)
   High risk: 63 studies (67.7%)
   Unclear: 31 studies (33.3%)
• Domain 2: Index test (AI/Deep learning model).
   Risk of bias
   Low risk: 0 studies (0.0%)
   High risk: 50 studies (53.8%)
   Unclear: 44 studies (47.2%)
• Domain 3: Reference standard
   Risk of bias
   Low risk: 41 studies (44.1%)
   High risk: 0 studies (0.0%)
   Unclear: 53 studies (56.9%)
• Domain 4: Flow and timing
   Risk of bias
   Low risk: 69 studies (74.2%)
   High risk: 0 studies (0.0%)
   Unclear: 25 studies (26.8%)
• The summary rating of 94 studies that were included was found to be the following:
   Low overall risk: 3 studies (2.2%)
   Moderate risk: 64 studies (68.8%).
   High overall risk: 27 studies (29.0%)
• Major concerns identified:
   Patient selection (risk of bias): 67.7% of the studies were high risk, mainly because they were retrospective using publicly available datasets.
   Index test (risk of bias): 53.8% was rated as high risk, presumably because of threshold optimization on test data.
   Reference standard: Generally well conducted (44.1% low risk, 55.9% unclear)
   Flow and timing: Best domain with 74.2% low risk rating.

## 4. Discussion

### 4.1. AI

AI has become a powerful tool for finding and treating DR, which is one of the most common causes of vision loss around the world. AI systems that use advanced algorithms, like deep learning models, can analyze retinal images with very good accuracy. This could be used to bring forth problems at an early stage to detect and treat. The yearly developments allowed the feedback of AI‐based screening technologies that may operate independently of human control. The first AI‐based diagnostic system that the U.S. Food and Drug Administration (FDA) approved of, though currently known as LumineticsCore, is the IDx‐DR system. It examines the retinal photos captured using a fundus camera and renders its findings immediately, allowing primary care physicians to identify the individuals requiring the attention of a specialist [102].

Moreover, in 2024, the FDA approved the AEYE Health AEYE‐DS system, which can be used with the Optomed Aurora handheld fundus camera [103]. This portable device, which allows the operator to take and analyze retinal images within a minute, can screen independently and in a short time (less than 1 minute) to detect DR in a large range of locations, including resource‐constrained areas [103].

It has been shown through numerous studies that AI has been effective in detecting of DR. The assessment of 15 studies and more than 150,000 images demonstrated that the average sensitivity of AI algorithms to diagnosing DR in retinal fundus photos was 92.58%, and the average specificity reached 87.22% [104]. A further meta‐analysis found that the pooled sensitivity of the AI systems was 87.7% with a specificity of 90.6% in some instances; this was superior to the results that general ophthalmologists could achieve [105].

The AI technologies are under consideration regarding their potential application in predicting the ways in which diseases will develop and in creating specific treatment plans. New solutions, like semisupervised graph ML algorithms, have recently came up to demonstrate that they can enhance the precision of DR identification based on labeled and unlabeled data [106]. In addition, the insufficient data issue can be solved by creating false retinal images via such techniques as StyleGAN3, as this synthesizes datasets to train and enhance the performance of the models, particularly of early stage DR detection [107].

These advancements notwithstanding, there is still no easy time to employ AI in the screening of DR on a large scale. To ensure that AI models can be applicable in many scenarios and are stable, it is necessary to address such aspects as the necessity to complete their testing under a variety of clinical conditions, variability in patient demographics, and variability in image quality [108]. There is also a need to note that AI‐based DR screen program success is reliant on their adaptability to the existing healthcare systems, their ethical correctness, and their cost [108].

### 4.2. Traditional ML in DR Detection

Conventional ML was very instrumental in the initial days of system development to detect DR automatically. These approaches most of the time incorporate a two‐step technique: They extract features on retinal images first and then use algorithms such as support vector machines (SVMs), random forests, or logistic regression to classify the images. The removal of some of the elements includes texture analysis and vascular segmentation, as well as other handmade descriptors depicting the appearance of DR‐linked lesions [109].

Despite these improvements, standard ML approaches have numerous problems, such as scaling and the use of many data types. The handcrafted features may drop performance on photographs of different qualities or groupings. Such strategies can also not be used to pick DR, especially at its initial stage, when the lesions are small in size. Classic ML methods remain useful, particularly when there are not enough computers or when understanding the model is of central importance. They are being developed as part of the research to compound them in a deeper learning configuration and develop more powerful DR detectors [110].

### 4.3. Deep Learning Models in DR Detection

Deep learning transformed how medical images are processed, and it has significantly impacted the detection of DR. Convolutional neural network (CNN) deep learning models can automatically discover significant features in large pools of retinal fundus imaging. This does not resemble common ML, which relies on handmade features. Given their ability to learn end‐to‐end, deep learning models are able to track down elusive, complex patterns in photographs that human doctors or conventional algorithms may have a hard time detecting. They must never forget [111].

The big datasets and many labels are required to ensure that deep learning models work well. The examples of public datasets that have been of great assistance in developing models include Kaggle EyePACS, the Asia‐Pacific Tele‐Ophthalmology Society (APTOS), and Messidor. Such databases often contain hundreds of fundus images, which ophthalmologists have tagged according to the severity of the DR. To gain more generalization and combat overfitting, human users frequently exploit image data augmentation techniques such as rota‐tilting (rotating), and flip (flipping), and altering the contrast [111].

The usual measures of deep learning models in DR detection are accuracy, AUC‐ROC, sensitivity, and specificity. The best models tend to reach an AUC of 0.90 and sometimes exceed the scores of human screening [111]. The regulatory‐approved deep learning systems, IDx‐DR and EyeArt, have been validated in substantial clinical trials [112]. The systems are efficient at analyzing retinal images and providing diagnostic advice without an evaluation by an ophthalmologist. Thus, they are appropriate in remote or underprivileged locales.

Raw data can be used by deep learning models to learn. However, that poses additional challenges, such as how this makes it difficult to interpret and how this is more likely to be one‐sided in training data. In order to remedy this, implementations of explainability tools such as Grad‐CAM and saliency maps that indicate the regions selected by the model as most critical in its predictions have been introduced [113]. Such images are useful in creating confidence to the doctors and in obtaining regulatory permission. Scientists also investigate new models where attention and vision transformers (ViTs) are applied to enhance the performance and make findings more comprehensible [114].

The state‐of‐the‐art on automated DR detection has become deep learning. Its capacity to process large, complex datasets, and obtain deep, tree‐like qualities seems to make it very suitable for the task. The ongoing development of model architecture, interpretability, and empirical replicability is supplementing the deep learning use in diabetic ophthalmology [111]. Some of the typical deep learning architectures and approaches employed in the incorporated studies are given below.

#### 4.3.1. CNN

CNN is the most common type of architecture for detecting DR. CNNs use several layers of convolution and pooling to process pictures. In so doing, they develop to know to identify edges, textures, and, at the higher level, microaneurysms, hemorrhages, and exudates, which are significant indications of DR. For DR classification tasks, popular architectures such as InceptionV3, Residual Network (ResNet), and EfficientNet have been trained and fine‐tuned a lot [115]. Also, AlexNet and ResNet‐101 were used as feature extractors for DR severity classification [1]. Some models can only predict two outcomes (referable vs. nonreferable DR), whereas others can predict several outcomes that show the disease is bad at different stages [116].

#### 4.3.2. ResNets

ResNets are an architecture of neural networks that have been presented in order to tackle the issue of training extremely deep modules. They may also be called (skip connections). They have been suggested in the study conducted in 2015 by Ref. [117]. ResNets have become a baseline architecture in deep learning, especially in computer vision. Some examples of RestNets are introduced below.

##### 4.3.2.1. The ResNet‐50 Model

Lin and Wu [9] examined the effectiveness of DR grading provided by updated ResNet‐50 and other commonly used CNNs, including AlexNet, Xception, VggNet‐s, VggNet‐16, and ResNet‐50. His results show the newly updated ResNet‐50 performs better against other popular CNNs when trained and tested on the Kaggle dataset (train accuracy: 0.8395 and test accuracy: 0.7432). The reason of that the novel structure of ResNet‐50 can prevent overfitting, minimize the loss value, and diminish the fluctuation issue.

Two‐phase research showed how a system based on a modified RestNet‐50, pretrained with the ImageNet data, could be used to categorize five ocular diseases, including DR, DME, cataract, and glaucoma. The model achieved a high specificity, sensitivity, and accuracy, which decreased the epochs and times of detection. A multifundus retinal image‐trained modified RestNet‐50 and DenseUNet model was utilized in the second phase to achieve an extreme specificity of 99.73%, a sensitivity value of 99.54%, and an overall accuracy of 99.67% [118].

##### 4.3.2.2. ResNet‐18 Model

According to one of the recent studies [10], the ResNet‐18 architecture with the Swish activation type has established itself as the best deep learning classifier in the detection of DR. The model was validated using the APTOS Kaggle database and used to analyze the actual data of the hospital. The proposed ResNet‐18 model with swish operation possesses a sensitivity of 93.42, an accuracy of 93.51, a precision of 93.77, and an F1 score of 93.59.

#### 4.3.3. Inception and Efficient Networks

Architectures of CNNs, such as Inception and EfficientNet, enhance the performance of deep learning models in achieving speed and accuracy. The first Google inception network was Google Net. To efficiently capture multiscale features, these networks use parallel convolutional routes with different kernel sizes in modules [119]. Later versions improved with factorized convolutions and residual connections. The EfficientNet compound scaling method developed by Google can adjust the depth, width, and resolution of networks by scaling coefficients. The family uses a lightweight base model (EfficientNet‐B0) that aims at producing a state‐of‐the‐art level accuracy using even fewer parameters and computation than with most CNNs. Real‐life implementation of the two concepts has led to increased expectation of performance and resource efficiency inflation [120]. Examples of inception and efficient networks are outlined below.

##### 4.3.3.1. Efficient DenseNet

Pravin et al. [14] classified the severity of DR using the suggested Efficient DenseNet. The results showed that Efficient DenseNet has a high test accuracy of 98.40% when coupled with a K‐NN classifier. The train and test were done on 13,000 retina fundus images, which are publicly available online on Kaggle.

##### 4.3.3.2. EfficientNetB3

Recently, Dixit & Jha [20] have demonstrated that the proposed strategy of labeling DR with the assistance of an EfficientNetB3 model and a squeeze and excitation block that is capable of classifying DR effective as it could award 88.44% accuracy, 98.00 percent specificity, 84.00 percent precision, 83.00 percent sensitivity, and 83.00 percent F1 score in the detection of DR based on five‐class fundus images of the APTOS 2019 dataset.

##### 4.3.3.3. Inception V3

InceptionV3 is a particular architecture of CNNs applied in establishing possible deep learning in the classification of images into their respective categories [121]. The model was trained using 8, 128, and 512 parameters, and after 170 epochs, it showed a good capability to capture features. The experiment by Pan et al. [122] used the ensemble of Inception V3 and ResNet‐50 as an approach to classifying fundus images. The results showed a strong model showing the best performance in the process of DR classification, getting the highest accuracies of 93.81 by ResNet‐50 and 91.76 by Inception V3.

#### 4.3.4. Ensembles of CNNs

It is possible to combine several CNN models to make improved forecasts, reduce the chances of overfitting, and strengthen the model. It is referred to as a CNN ensemble. The point is that both models can possibly produce various errors in respect to generalization, but the combination with each other can resolve their deficiencies in the way of averaging, voting, or stacking. This method is very beneficial for applications that need a lot of accuracy, such as classifying images, evaluating medical images, and finding objects [121]. An example of an ensemble of CNNs [7] revealed that combining five deep learning models, including CNN, EfficientNet, VGG 16, ResNet‐50, and MobileNet, by adjusting the settings of these models has made DR classification more accurate.

#### 4.3.5. Transfer Learning

In some works, it has been observed that transfer learning with pretrained models on generic image datasets like ImageNet has dramatically enhanced performance, particularly when labeled medical data are scarce [123]. In the recent past, this approach has begun to be recognized as a gold standard. According to Yang et al. [25], ResNet‐50 models trained on the ImageNet dataset and using the convolutional block attention module (CBAM) classifier recorded an accuracy of 94.4% with the pretrained model. Another study was performed by Shimpi and Shanmugam [124]. In this approach, the VGG16 pretrained model with adaptive boosting techniques was used on CNN‐based classification models and showed much better precision and area under the curve (AUC). Transfer learning significantly improves the convergence rate, and frequently the performance, especially in situations where there is a small training set.

#### 4.3.6. Generative Adversarial Networks (GANs)

The problem with class mismatches and insufficient training data can be addressed with GANs. GANs can make realistic fake fundus pictures showing different DR lesion patterns, adding rare cases to the dataset. This makes CNNs stronger and helps them learn better. GANs have also been used to improve the quality of pictures (noise reduction or super‐resolving retinal images) and even find problems by learning what a normal retina looks like [125].

#### 4.3.7. Recurrent Neural Networks (RNNs)

A type of artificial neural network called a RNN is made to process sequential data. In contrast to conventional feedforward neural networks, RNNs are able to retain some sort of memory over prior inputs because of their connections, which create cycles. This is especially useful in an activity where context or order of information is relevant [126].

#### 4.3.8. Attention Mechanism

A new method has been published recently to help the model focus on important retinal areas (microaneurysms, hemorrhage areas). Instead of treating all inputs the same, they let a model focus on the most important parts of the input as it makes predictions. This kind of method, like ViTs [127]. According to a recent study by Romero‐Oraá et al. [15], focusing on the bright and dark pixels in the retinal image employing a different attention mechanism enables the development of separate attention maps for red and bright lesions. The results showed that the separate attention approach has been successful at improving the classification.

### 4.4. Clinical Application Models and Real‐World Deployment

Recently, the use of AI for DR screening and diagnosis has become common in outpatient clinics, while before it was just limited to lab work. This led to several screening systems that functioned perfectly. These AI models are mostly based on deep learning methods. which has undergone clinical validation and is used to detect DR in practice, running without immediate expert control [128]. Major examples have been discussed below, and how they perform in real‐life contexts.

#### 4.4.1. IDx‐DR (Digital Diagnostics’ LumineticsCore)

Digital Diagnostics Inc. produced IDx‐DR, which is now called LumineticsCore. It is an FDA‐approved AI system that can find DR in adults with diabetes on its own. It was the first AI diagnostic tool that could make a medical conclusion without any human help [129]. It can be used in primary care settings to look at retinal images taken by a suitable camera and find indicators of more than mild DR. The results are ready in just a few minutes. Its clinical accuracy was high, and its sensitivity and specificity were 87.4% and 89.5%, respectively [130]. Another study done in the Netherlands in Ref. [131] found that sensitivity was between 79% and 91% and specificity was between 85% and 94%, depending on the grading standard. The FDA’s approval of it was a huge step forward for AI in healthcare because it made it easier to spot early indicators of diabetic eye disease [129].

#### 4.4.2. EyeArt (Eyenuk Inc.)

Another AI system for self‐directed DR screening is called EyeArt, and it was created by Eyenuk. It was approved by the FDA in 2020 for the detection of both more‐than‐mild and vision‐threatening DR, and it was CE marked in Europe. Hundreds of thousands of patient photos have been used to certify EyeArt. A comprehensive real‐world study done by Bhaskaranand et al. [132] included more than 100,000 primary care screening visits; the EyeArt v2.0 system identified referable DR with 91.3% sensitivity and 91.1% specificity. Another study that looked into EyeArt’s sensitivity in screening situations found that it was much higher than that of conventional ophthalmologists. The performance and methodology of EyeArt make it a valuable tool to screen DR in scale. It has been used in programs in many different nations [133].

#### 4.4.3. Google AI/Verily Deep Learning System

Many clinical studies have investigated and approved the use of the Google algorithm in the previous 5 years. The first study to prove that deep learning models can accurately diagnose DR was Gulshan et al.’s study conducted in 2016. In 2022, another study in Thailand was done by Ruamviboonsuk et al. [134]. Considered a Google deep learning system as a solution for real‐time screening, it proved to be a successful AI tool for analyzing fundus images. It was able to find a referable DR with an accuracy of about 95%, with 91.4% sensitivity and 95.4% specificity compared to the reference standard. This is similar to the performance of ophthalmologists who looked at the same dataset and found 84.8% sensitivity and 95.5% specificity under the study’s conditions. This means the model can adapt to changing conditions and screen like an expert. Google’s solution is not commercially available, but it shows how a research model is validated on patients. The Thailand study, as a human–AI collaboration, puts a lot of emphasis on workflow integration [134].

#### 4.4.4. Economic and Public Health Perspectives on Scaling AI System Solutions

From a public health and economic standpoint, AI‐enabled DR screening has the potential to enhance accessibility and cost‐effectiveness, especially in resource‐limited settings where shortages of trained ophthalmologists impede regular screening. Several economic evaluations, including cost‐effectiveness analyses, suggest that AI‐based screening strategies can reduce per‐person screening costs and improve quality‐adjusted life years (QALYs) compared with no screening or traditional manual grading approaches, although the degree of economic benefit varies by context and program design [135]. Some models have found AI screening cost‐effective under accepted willingness‐to‐pay thresholds, while others emphasize that maximizing cost‐effectiveness may require balancing sensitivity and specificity trade‐offs and ensuring patient referral adherence. By reducing reliance on specialist graders and lowering screening costs, scalable AI systems could support population‐level early detection initiatives, reallocate healthcare resources more efficiently, and expand equitable access to DR screening in underserved regions [135]. The cost‐efficiency of AI‐based DR screening can vary even in the same country, depending on the perspective used. Indicatively, in comparison with urban settings that had easier access to treatments, transportation cost, which is a direct nonmedical cost, played a significant role in determining the cost‐effectiveness of DR screening in rural China [136]. Patients in remote areas could find the challenge of getting to a medical facility to be a big problem [137].

### 4.5. Performance Metrics and Evaluation

#### 4.5.1. Accuracy

One performance indicator in the assessment of AI systems in the detection of DR entails the measurement of accuracy. It indicates the general proportion of the correct prediction of the model in terms of true positives and true negatives. It is a broad panel on the diagnostic efficiency of the system. The accuracies are very high (> 90%) in the most recent studies. However, sensitivity and specificity deserve to be seen too [102].

#### 4.5.2. Sensitivity and Specificity

The key performance metrics that are vital in the evaluation of the performance of AI systems in detecting DR include sensitivity and specificity. Sensitivity or true positive rate refers to the success of the system in getting the right cases (DR patients) so that it does not miss any case. Specificity, or the true negative rate, evaluates the performance of the system in limiting the instances of false positive identifications of persons not in the possession of the disease and subsequently identifying those that have the disease as such. Most of the existing AI models are those that are highly sensitive. In the case, the IDx‐DR trial, where sensitivity was prioritized as a patient safety outcome, over 87% sensitivity was achieved at the rate of sample specificity of ∼91% [131]. In the same manner, a trial of the research model by Google produced a sensitivity exceeding 91% and a specificity exceeding 95%, and the EyeArt system also reached a compromise between sensitivity and specificity at almost 91% [132, 134]. These are higher as compared to screening with general ophthalmologists, where sensitivity ranged between 73 and 85% in Abràmoff et al. [130]. Some of the more prominent deep learning models purported sensitivity rates up to 95 99% [138].

#### 4.5.3. Precision and F1 Score

Accuracy and F1 score are regarded as the core values of AI models diagnosing DR. Precision (also known as positive predictive value) is used to verify the correctness of a positive diagnosis by measuring the proportion of real positive outcomes among all positive model predictions [139]. Precision is likely to be worse in DR screening than sensitivity, since the prevalence of true DR is fairly rare, and a highly sensitive model will classify many instances (some of which are false positives). For example, when sensitivity is high, an automated system may only have ∼30% precision, which means that many referrals are preventative [131].

The F1 score is one measure that incorporates both precision and recall since it is the harmonic mean of the two. F1 is commonly mentioned by researchers when they intend to measure the whole effectiveness of the classification, especially when the classes are unbalanced. Strong performance is demonstrated by the fact that many recent studies indicate F1 scores for their CNN models within the 0.8–0.9 range [140].

#### 4.5.4. AUC

AUC is a necessary parameter when it comes to assessing the total diagnostic efficiency of the use of AI models in the diagnosis of DR. It is a common device applied to the DR studies to assess the model performance indicators across sensitivity and specificity. A high AUC indicates that, with the right thresholding techniques, the model can achieve high sensitivity and specificity [141] in their validation datasets study, the most advanced deep learning models for DR have shown AUCs of approximately 0.95–0.99. While the Google CNN, for example, demonstrated outstanding discriminative ability with an AUC of 0.991 on EyePACS datasets and 0.990 on Messidor‐2 datasets. AUC > 0.90 on internal test sets is reported by the majority of well‐trained deep models in the recent literature [141].

#### 4.5.5. Real‐World Validation

Results in the real world may not always match performance in controlled test sets. Thus, future demonstration and external testing are vital measures of success. The majority of the newly established automated AI products, such as LumineticsCore (formerly) and the implementation of EyeArt in the clinic, have been tested in the real world as a way of verifying their performance, stability, and clinical usefulness beyond the limits of research trials. These studies regularly show sensitivity and specificity in the high 80s or 90s, despite frequently using slightly different devices (such as detecting “more‐than‐mild DR” or “referral‐warranted DR”) [132]. Due to the use of large populations (over 100 patients for EyeArt and multiple sites for IDX and Google), the results verify that AI models continue to maintain performance outside the lab. However, some cases show low performance in certain sittings, for instance, if the patient population exhibits unusual traits or if the images are of lower quality. For AI systems to continue to be dependable in a variety of healthcare settings, ongoing postmarket monitoring and improvement (continuous learning) are critical [128].

### 4.6. The Effect of Dataset Size on Deep Learning Performance

**FIGURE 2:**
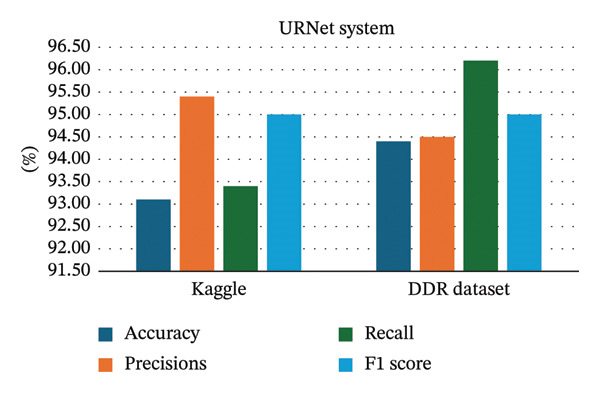
The performance metric for the URNet system model using different datasets.

**FIGURE 3:**
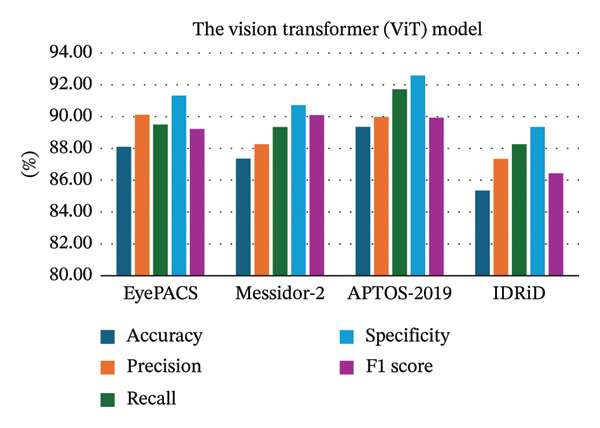
The model’s high‐performance metrics compared to different datasets.

**FIGURE 4:**
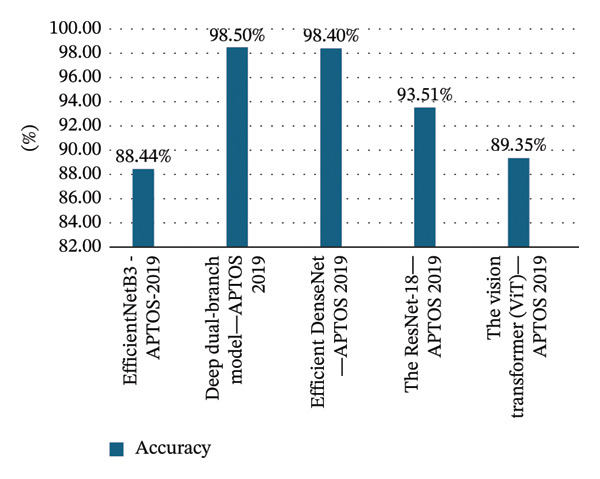
Different models’ accuracy with the same datasets.

Deep learning models are trained to identify patterns in large volumes of data. The general idea behind the large data is that it provides more different and subtle representations to the model to learn, and thus, it will achieve better performance [142]. Where is the bigger dataset is giving the more examples to learn. This will reduce the likelihood of overfitting and enhance its capacity to extrapolate to the unobservable data [142]. For example, Yang et al., [25], in their study about the URNet system, utilized ResNet‐50 as a pretrained model. When the model was trained and tested by a larger dataset, the DDR dataset, which has 13,673 fundus images, the performance metrics were higher than when using the Kaggle dataset that has 5100 images, as seen in Figure 2. Another example, a study done by Zhang and Chen, [22], about the ViT model, when trained by the EyePACS dataset, shows different performance metrics compared to when using APTOS2019, Messidor‐2, and the IDRiD dataset, where the performance metrics were highest on the APTOS 2019, followed by the EyePacs datasets, as seen in Figure 3. However, models such as the ViT, EfficientNetB3, deep dual‐branch model, Efficient DenseNet, and RestNet‐18 show different accuracy range using the same datasets (APTOS‐2019). Where is the top highest model was a deep dual‐branch model (98.50%), followed by EfficientDenseNet (98.40%) as seen in Figure 4. Study conducted by Ref. [143] in the classification of DR in terms of transfer learning using CNN‐based models, the paper has given special attention to InceptionResNetV2 as the most successful model, as it trained the models with a precision of 82 on the test. They mention that the performance of the deep learning models can be refined and can be more precise when a large amount of trained data are used.

### 4.7. The Geographic Location of the Included Studies Pertaining to AI Models for DR

**FIGURE 5:**
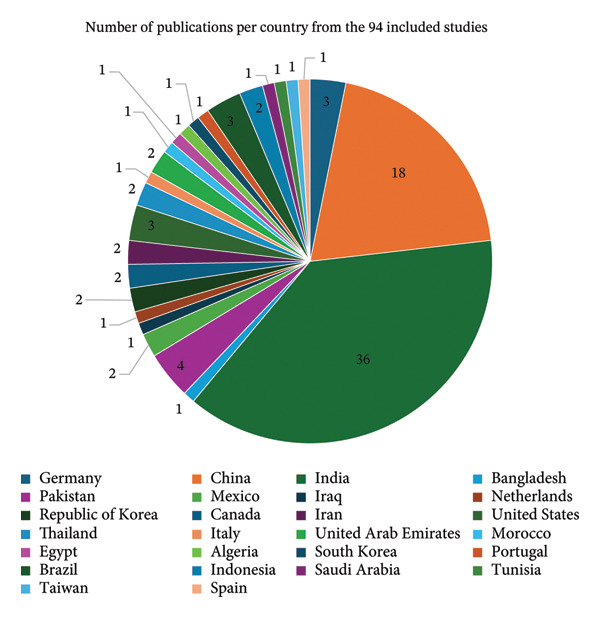
The number of publications per country from the 94 included studies.

Figure 5: shows that among 94 studies included in the review, 36 were published by Indian researchers, indicating that India had the highest contribution of publications regarding DR detection and classification. The high contribution of India highlights the fast‐growing Indian research and institutional participation across various disciplines. The studies that were published in India were aimed at detecting of DR. Mostly, they examine the deep learning models, particularly transfer learning, ResNet, DenseNet, EfficientNet, ResNet with DenseNet and EfficientNet, a metalearning approach, multiclass adaptive boosting approach, superlearning, coordination attention mechanism, DR‐XAI: explainable deep learning Model, and other variants of ensemble deep neural networks. To illustrate the points, Sunkari et al. [10], Vij and Arora [80], and Sangeetha et al. [144] showed general overviews and diagnostic systems based on transfer learning by Sunkari et al. [10] also proposed a change to ResNet‐18 and its activation (Swish). The work of India covers several areas of the DR field: feature extraction, image segmentation, classification, ensemble learning, and explainability (DR‐XAI by Vasireddi et al., [145]). Its quantity alone points to clinical importance as well as the high academic activity.

The results depicted that China contributed the second‐highest number of publications, comprising a total of 18 publications. This means that China has maintained the investment in research and technologies. The studies published by China pay so much attention to advanced means of imaging, lightweight, and contrastive learning. Among innovations, it is important to mention the application of nonmydriatic photography‐assisted telemedicine [97] and the prediction based on OCT‐based nomograms [146]. It also emphasizes cost‐effectiveness and community screening [92, 25], semisupervised models [92], Cascaded context fusion, and a multiattention network [94]. The body of work developed by China demonstrates a unified theoretical approach, a combination of clinical relevance and sophistication of algorithms.

Pakistan has contributed with four publications, coming as the third after India and China, the Pakistani publications dealt with various topics. For example, Pakistani scholars studied the use of CNNs in one of the largest ophthalmology institutes [11], whereas Alsadoun et al. [98] discussed AI‐powered fundus analysis. While Ashraf et al. [43] discussed the used of deep red lesion classification by using the deep CNN model for DR diagnosis. Lastly, Ullah et al. [46] study has utilized genetic algorithm–based CNN features by using five CNN architectures extract features: AlexNet, NASNet‐Large, VGG‐19, Inception V3, and ShuffleNet. Such contributions posit that more interest emerges in using AI for DR in clinical contexts, especially in the context of public health infrastructures.

The United States, Brazil, and Germany each contributed with three publications each; the U.S. studies discussed different aspects such as the use of deep learning techniques for DR screening, deep learning frameworks with smartphones for the detection of DR, and the use of ChatGPT‐4 for DR grading. While Brazilian studies discussed detecting fundus lesions by using CNN, image processing, and deep neural network, they also used red lesion localization and CNNs. The German studies had addressed some of the deep learning approaches, deep kernel learning methods on DR grading and deep feed forward neural network–based screening system for DR severity and evaluated CNNs, ViTs, and hybrid models for DR classification.

Moreover, countries such as Canada, Mexico, Iran, the Republic of Korea, the United Arab Emirates, Indonesia, and Thailand each contributed two publication studies, indicating their role in research operations. The research that was published in these countries paid attention to other aspects. In Thailand research, the light is in the lesion segmentation, such as the determination of hemorrhage and exudates by lesion segmentation methods [17, 78]. These papers involve not only the application of deep learning but also conventional image processing (e.g., watershed processing), which provides useful insights and solutions in the case of early stage DR detection. Whereas Canadian publications focused on biomarkers and validation studies, one took a look at a noncoding RNA panel as a biomarker [85] and one on the prospective validation of an AI‐based DR screening system at a Quebec hospital [77]. Such works are a representation of clinical interests and molecular diagnostics interests. While Mexican studies highlighted the use of deep learning in the classification of retinal fundus images for DR and computer‐aided diagnosis of diabetic retinopathy lesions.

Besides, some nations such as Bangladesh, Iraq, the Netherlands, Italy, Morocco, Egypt, Algeria, South Korea, Portugal, Tunisia, Taiwan, Spain, and Saudi Arabia have contributed with fewer publications, as each of them had only one publication. This suggests a much lower level of interest in the subject when compared with India and China. The contribution of these countries was focused on different aspects, such as a study globalized in Iraq by Omer [93], which posed a bilayered neural network based on resubstitution validation strategies. In contrast, the one completed in Germany through Siebert et al. [6] carried out an uncertainty analysis on advanced deep kernel learning strategies. A study of Iran done by Shakibania et al. [8] proposed a new architecture to grade levels of DR, and research conducted in Taiwan by Lin and Wu [9] made changes to the ResNet‐50 to improve DR detection. Khan et al. [99] have published a study conducted in Bangladesh, which introduced a computer‐aided diagnosis system based on transformers with an emphasis on low‐resolution imagery. Romero‐Oraá et al. [15] proposed an attention‐based framework in grading fundus images in their Spanish study; the Indonesian study by Hendrawan et al. [16] developed ensemble CNNs in DR classification, the Mexican study by Moya‐Albor et al. [95] applied knowledge distillation in lesion diagnosis, and the Portuguese study by Monteiro [19] used a blended deep learning approach in grading. However, the Netherlands study done by Diware et al. [90] focused on the screening using memristor‐based neural networks. Lastly, the South Korean study by Kim et al. [86] used transformer‐enhanced segmentation with multiscale fusion.

**TABLE 5:** Performance of notable autonomous DR screening AI systems.

AI system (study)	Sensitivity (referable DR)	Specificity (referable DR)	Study details and notes
IDx‐DR [130]	87.2% (95% CI ∼81.8%–91.2%)	90.7% (95% CI ∼88.3%–92.7%)	U.S. prospective multicenter primary care trial. Initial FDA‐approved autonomous DR diagnosis. Achieved superior outcomes (> 85% sens, > 82.5% spec). Great imageability (96% of patients got results)
EyeArt [132]	91.3% (90.9–91.7)	91.1% (90.9–91.3)	Retrospective analysis of ∼101k patient visits (404 clinics). Auto‐DR screening on the cloud. Very sensitive over a wide, varied population. Sensitivity ∼98.5% for vision‐threatening DR
Google AI [134]	91.4% (87.1–95.0)	95.4% (94.1–96.7)	Potential deployment in nine Thai primary care settings. AI vs. retinal graders. AI had same specificity and higher sensitivity than specialists, proving national screening program viability
Human Graders [134]	84.8%	95.5%	Comparison of experienced ophthalmologists/retina specialists in Thailand. Shows that AI can meet or exceed human screening sensitivity

## 5. Summary of the Findings

• The findings reveal that AI models have contributed significantly to the early detection of DR. Traditional ML was largely ineffective, with low success rates using expert‐constructed features. The past 5 years of deep learning have moved performance to new levels and have become able to achieve near‐expert accuracy in the detection of referable DR based on fundus photographs in the real world. The finding also indicates that models, such as ResNet‐based CNNs frequently augmented with transfer learning and ensembling, may achieve sensitivity and specificity above 90%, as reflected in research tests and clinical trials.
• The URNet system, utilizing ResNet‐50 and the DDR dataset, achieved 94.5% precision and high recall rates for NRDR and RWDR. The VGG16 model with Kaggle data achieved 91.6% accuracy and a kappa score of 0.883. ResNet‐50 and EfficientNetB0 models with the APTOS 2019 dataset reached an accuracy of 98.50%, with high sensitivity and specificity. The DenseNet model using the Kaggle dataset achieved 98.40% accuracy. The ResNet‐18 model demonstrated 93.51% accuracy and other strong performance metrics using the APTOS Kaggle database.
• The size of the dataset affects the performance metric of the AI models. For example, the ViT, when trained by the EyePACS dataset, it shows different performance metrics compared to when using a large dataset, such as APTOS2019, Messidor‐2, and the IDRiD dataset, where the performance metrics were highest on the APTOS 2019, compared to other datasets.
• The autonomous systems described in Table 5 demonstrate that such models are not merely exercises in the field of study but are now moving closer to becoming effective screening tools. The sensitivity and specificity, as well as the AUC values, are high, indicating that AI can be used with a high degree of confidence to detect early DR, and this accuracy is important in identifying vision loss.
• Autonomous DR screening devices, like ADX‐DR, have shown commendable performance. High imageability was observed, with 96% of patients receiving results. The EyeArt modality exhibited exceptional sensitivity across diverse populations, detecting ∼98.5% of vision‐threatening DR. Google AI matched specialist performance in specificity and surpassed them in sensitivity, validating the national screening program’s viability.
• The findings show that Asian countries, including India and China, have the largest contribution of publications regarding the use of AI models, specifically deep learning and ML models for the detection and classification of DR. This indicates the interest and development of the research activities in these countries and reflects their bigger investments in scientific research and technology. However, the findings also indicate a few of contribution to research and publication in African countries or low‐income countries, especially in the north of Africa and the Middle East countries. It is recommended that future research on DR studies needs to be conducted in African and low‐income countries to reduce the prevalence of DR, limiting diabetes complications that lead to blindness and visual loss.

## 6. Conclusion

The paper presents an in‐depth analysis on the application of AI models in the detection and classification of DR. Early detection of DR has been of great importance because of the assistance of AI models. Deep learning models can reach expert‐level accuracy in identifying referable DR in fundus photographs in real‐world situations, whereas in methodologies using traditional ML‐based methods, the success prior has been limited with expert‐created features. Deep learning, and specifically models based on CNNs, extended with transfer learning and ensembling consistently show a high sensitivity and specificity, which in research settings and clinical trials has been as high as over 90% in some cases. Numerous CNN‐based models demonstrated an outstanding contribution to DR detection in the past 5 years, often performing better than human accuracy. The sensitivity, specificity, and AUC of these AI models are very high, pointing out that AI can detect early DR with the highest level of confidence, which is important in preventing vision loss. Clinical validation of several AI systems (e.g., IDx‐DR and EyeArt) is complete, and, indeed, these AI systems pass the test in real‐life screening, which reinforces the high specificities and sensitivities of AI systems.

The autonomous AI has moved beyond being an academic exercise, and its increased use successfully screens and classifies DR. Their capability to work independently without direct input from an expert allows this technology to be implemented at a large scale, particularly in regions that have low access to ophthalmologists. The biggest contributors to the development of AI models in the detection and classification of DR are Asian countries, specifically India and China, where significant resources and investment into this field of research are taking place. On the other hand, limited input from African and low‐income countries is evident, and there is a need to conduct more studies on these domains to represent the numbers that are affected by DR, leading to the loss of vision.

In perspective, further gains are expected. New solutions include ensemble models, federated learning, and data augmentation using GANs, which have the potential to further increase performance and generalizability. More diverse data will be available, and already‐existing algorithms will be improved iteratively, so we can rely on AI‐based DR detection getting increasingly precise and reliable. Finally, the transformation of these models to practice, in the form of decision support among ophthalmologists, or fully autonomous in primary care, promises to help prevent diabetic blindness in thousands of cases, which is the desired objective that can be achieved: early diagnosis and treatment of DR. Overall, AI‐based models, and particularly deep learning, have transformed the approach to DR detection through high‐accuracy, reliable, and even self‐governing screening methods. Although the clinical potential of these interventions has been shown, there is an apparent need to increase research and implementation projects in underserved areas to address vision loss more efficiently.

### 6.1. Data Safety and Ethical Concerns

Ethical issues and data security in research necessitate the protection of the privacy of participants by encryption, anonymization, and access control measures to ensure that the information is not compromised. The essential ethical needs are to have informed consent, a sense of confidentiality, a sense of voluntary participation, and to limit harm. These issues are vital to research integrity, data prejudice, transparency, and data proprietorship. Although in this review study, the data do not involve direct human participation, it does not require ethical approval or a consent form for participation. However, studies included in this review utilized publicly available online data using fundus eye images to classify DR severity.

### 6.2. Limitations of the Study

Although the study aimed to provide and discuss a comprehensive review of the use of AI models in the detection of DR by conducting a comprehensive search of databases and including 94 studies, many recent studies were excluded from the review due to the unavailability of the full text. Also, most of the studies utilized publicly available data, which was limited to study results, which may affect the generalizability of results. Furthermore, there were limited publications and studies in Africa and low‐income countries, which may increase concerns about screening and selection bias, as well as raise the need for additional investigation on the use of AI models in screening for DR [67].

## Funding

The authors have nothing to report.

## Conflicts of Interest

The authors declare no conflicts of interest.

## Data Availability

All data generated or analyzed during this study will be available on request.

## Appendix A Characteristics of Included Studies

**Table A1:** Sixty‐one included studies with deep learning approaches.

	Article name and citation	Model approach	Datasets	Performance metrics	Strengths and weaknesses of the study	Study location
1	Uncertainty analysis of deep kernel learning methods on diabetic retinopathy grading [6]	The model uses deep kernel learning (DKL) for uncertainty estimates. DKL combines EfficientNet‐B0 with a Gaussian process layer	Kaggle Diabetic Retinopathy Detection (EyePACS) database. Indian Diabetic Retinopathy Image Dataset (IDRiD). Retinal Fundus Multi‐disease Image Dataset (RFMID). Society for Imaging Informatics in Medicine/International Skin Imaging Collaboration (SIIM‐ISIC) dataset	It is not addressed in the paper	The study highlights the model’s generalization ability for distribution shifts Deep ensembles significantly improve model performance and uncertainty quality. The study addresses miscalibrated uncertainties in neural networks. Results do not fully support previous positive findings	Germany
2	MCBM: Implementation of multiclass and transfer learning algorithm based on deep learning model for early detection of diabetic retinopathy [7]	The study employs automated deep‐learning techniques for DR screening	Kaggle’s Diabetic Retinopathy Detection APTOS 2019 dataset, consisting of 3662 retinal images. EyePACS dataset, referenced in related work	EfficientNet achieved a training accuracy of 0.9342 and a testing accuracy of 0.8181. ResNet‐50 achieved a training accuracy of 0.9329 and a testing accuracy of 0.8116. Average Top1 accuracy across models is 77% and Top5 accuracy is 93%	The study utilized a large dataset of 3662 images for training and testing EfficientNet and ResNet achieved the highest accuracy of 81%. Higher‐quality data may lead to discrepancies in results. Publicly available data limited some studies’ results	India
3	Dual‐branch deep learning network for detection and stage grading of diabetic retinopathy [8]	The model utilizes transfer learning for feature extraction. It employs ResNet‐50 and EfficientNetB0 as pretrained models	APTOS 2019 dataset. Messidor‐2 dataset. IDRiD dataset. EyePACS dataset. Messidor dataset (relabeled version)	The model achieved an accuracy of 98.50% for binary classification. Sensitivity was reported at 99.46% and specificity at 97.51%. Quadratic weighted kappa score was 93.00% for stage grading. The model’s accuracy for stage grading was 89.60%. AUC scores were 98% for binary and 90% for multiclass classification	The model achieved high accuracy and sensitivity in diabetic retinopathy detection . Image augmentation techniques enhanced the model’s generalization capabilities. Limitations exist in distinguishing severe and proliferative diabetic retinopathy case Future research may improve model performance through advanced techniques	Iran
4	Development of revised ResNet‐50 for diabetic retinopathy detection [9]	The study proposes a revised ResNet‐50 model for DR detection	DR dataset from Kaggle, which includes 35,126 fundus images, of which 25,805 are normal (without disease) and 9321 exhibit diabetic retinopathy (DR)	The averaged accuracy of DR grading is 0.9485	Strength: Revised ResNet‐50 reduces overfitting and loss value. Adaptive learning rate enhances model performance. Effective preprocessing improves image quality and accuracy. Weakness: Limited external funding may affect resource availability. Potential data variability from different fundus photography equipment	Taiwan
5	A refined ResNet18 architecture with Swish activation function for diabetic retinopathy classification [10]	The model utilizes the ResNet‐18 architecture with Swish activation function	APTOS Kaggle database. Clinical dataset provided by Sankara Nethralaya (SN) and MDRF (Madras Diabetic Research Foundation). IDRID dataset	The proposed model achieved 93.51% accuracy in classification. Precision was reported at 93.77% for the model. Recall was measured at 93.42%. The F1 score of the model was 93.59%	Strength: Achieved a high accuracy of 93.51% in DR detection. Utilized ResNet‐18 with Swish for improved performance. Comprehensive comparative study with various architectures. Weakness: Limited dataset of 5371 fundus images. Potential overfitting due to small training dataset size	India
6	A prospective study on diabetic retinopathy detection based on modify convolutional neural network using fundus images at Sindh Institute of Ophthalmology & Visual Sciences [11]	A modified convolutional neural network (CNN) was utilized	Testing dataset from patients’ data at Sindh Institute of Ophthalmology & Visual Sciences (SIOVS), Hyderabad, Pakistan. Private dataset consisting of 57,625 diabetic retinopathy (DR) images, labeled either DR positive or DR negative	The model achieved 93.72% accuracy on the test data. Sensitivity was reported at 97.30%. Specificity was noted as 92.90%	The model achieved 93.72% accuracy in detecting diabetic retinopathy. High sensitivity of 97.30% indicates the effective detection capabilities. Utilized a private dataset for training and validation. Limited testing dataset collected from a single device. Requires large amounts of fully labeled data for training. Future improvements can include semilabeled and unlabeled dataset	Pakistan
7	A deep learning‐based transfer learning approach fine‐tuned for detecting diabetic retinopathy [12]	The model employs VGG16 architecture for feature extraction	Indian Diabetic Retinopathy Image Dataset (IDRiD)	The study achieved an accuracy of 90.4% in diabetic retinopathy classification	Achieved high accuracy of 90.4% in diabetic retinopathy classification. Utilized an advanced VGG16 architecture for feature extraction. Employed various machine learning methods for robust classification. Focused on a significant health issue affecting diabetics. Limited to Indian diabetic retinopathy image datasets. Future research needed for noise reduction and feature selection	India
8	A novel automated system for early diabetic retinopathy detection and severity classification [13]	A novel DL approach integrates multiple components for feature extraction. Col‐Net uses VGG16‐bn for extracting information from retinal images. Mobile Net V3 provides robust classification of DR severity levels	APTOS 2019 Blindness Detection dataset, DiaRetDB1 V2, Messidor‐2 dataset and DiaRetDB0 dataset	Accuracy measures correct identifications versus total images. Precision indicates correctly classified positive cases. Specificity refers to true negative classifications. Sensitivity measures correctly classified retinal images. F1 score averages precision and recall scores. IoU shows the region covered by identified and ground truth images	Strength: Achieves 99.63% accuracy in DR detection. Utilizes advanced deep learning techniques for feature extraction. Weakness: Lacks metaheuristic‐based fine‐tuning process. Limited interpretability of the model. Does not address data privacy and security issues	India
9	An efficient DenseNet for diabetic retinopathy screening [14]	The study proposes an efficient DenseNet model for diabetic retinopathy detection. The model includes a data augmentation unit and classification modules. It utilizes a k‐nearest neighbor classifier for improved accuracy	Asia‐Pacific Tele‐Ophthalmology Society (APTOS) image dataset, containing 5590 retinal images, which was augmented to 13,000 images for the study	The proposed model achieved a classification accuracy of 98.40%. High recall and specificity values were reported for severity grading. The framework demonstrated a precision rate of about 74.58%	The study proposes a novel deep learning framework, efficient DenseNet. Efficient DenseNet shows high accuracy in diabetic identification. Utilizes advanced feature extraction for improved classification. Comparison with baseline models demonstrates superior performance. Lacks detailed information on limitations or weaknesses	India
10	Attention‐based deep learning framework for automatic fundus image processing to aid in diabetic retinopathy grading [15]	The model uses an attention‐based deep learning framework	EyePACS retinal image dataset for diabetic retinopathy detection competition (Kaggle)—35,126 images for training and 53,576 for testing. A subset of 18,860 images used for training, 2096 images for validation, and 32,017 images for testing	The proposed method achieved 83.7% accuracy on the test set	The study achieved 83.7% accuracy in DR classification. It generated interpretable attention maps for lesions. Poor detection accuracy for Class 4 was noted. The image decomposition algorithm is time‐consuming. Low resolution of attention maps limits lesion detection	Spain
11	Classification of diabetic retinopathy using ensemble convolutional neural network architectures [16]	The study used ensemble convolutional neural networks (CNN) architectures	Indian Diabetic Retinopathy Image Dataset (IDRiD)—comprising 516 original color fundus images, including 168 normal fundus, 25 mild NPDR, 168 moderate NPDR, 93 severe NPDR, and 62 PDR images	The model achieved an AUROC of 0.88 for diabetic retinopathy detection. Sensitivity was reported at 0.89, with 65 true positives out of 73. Specificity was 0.90, with 27 true negatives out of 30	Strength: High sensitivity and specificity in DR detection. Utilizes ensemble CNNs for improved classification accuracy. Dataset available online for public access. Weakness: Limited to a small dataset of 516 images. May not generalize well to other populations	Indonesia
12	Deep learning‐based hemorrhage detection for diabetic retinopathy screening [17]	HemNet is a shallow network for hemorrhage classification. It contains 19 individual layers, including input and output. The model uses seed points for object segmentation	DiaRetDB1—contains 89 fundus images, with five being standard and the rest showing various retinal pathological symptoms. DiaRetDB0—contains 130 images, of which 110 have diabetic retinopathy signs	The proposed algorithm uses sensitivity, specificity, precision, and accuracy metrics. Area under the curve (AUC) is a critical evaluation criterion. The precision–recall curve assesses model performance. The algorithm’s performance is validated through synthetic experimentation	Strength: Automatic detection reduces screening time and complexity. Competitive accuracy compared to a renowned deep learning models. Noninvasive method enhances patient safety during diagnosis. Weakness: Increased network layers do not guarantee better results. Limited dataset may affect generalizability of results	Thailand
13	Diabetic retinopathy classification using deep learning [18]	The model employs a convolutional neural network (CNN) with ResNet‐152 pretraining. It uses transfer learning for classification on fundus images. The model was trained using the Adam optimizer and a learning rate of 0.001	Kaggle dataset of 3662 fundus photographs from patients diagnosed with diabetic retinopathy (DR). Asia‐Pacific Tele Ophthalmology Society’s 2019 Blindness Identification (APTOS 2019 BD) database	Performance metrics include accuracy, precision, recall, and AUC‐ROC. These metrics evaluate model performance in diabetic retinopathy detection. The model achieved an overall accuracy of 78.14%	The study achieved an overall accuracy of 78.14%. Utilized transfer learning for improved model performance. Outperformed recent cutting‐edge techniques in DR detection. Potential for early disease diagnosis and treatment in clinical settings. Deep learning models may lack interpretability in clinical applications. Requires larger, diverse datasets for better generalizability	India
14	Diabetic retinopathy grading using blended deep learning [19]	A blended deep learning model was proposed for DR grading. Ten state‐of‐the‐art deep learning networks were utilized	Indian Diabetic Retinopathy Image dataset (IDRiD)—516 images. Messidor‐2–1748 images. Asia‐Pacific Tele‐Ophthalmology Society (Kaggle APTOS) dataset ‐ 5590 images. Kaggle EyePACS ‐ 88,702 images. DDR dataset ‐ 13,673 images. Balanced DDR dataset (BDDR)—31,330 samples	The blended approach improved overall accuracy for DR grading. Averaging predictions achieved the best performance among methods tested. High accuracy was noted for normal retina and mild DR detection	Strength: Utilizes a blended deep learning approach for DR grading. Strength: Combines predictions from multiple models to enhance robustness. Addresses imbalanced datasets through balanced training. Weakness: Model reliability concerns due to imbalanced datasets in literature. Lower accuracy with balanced datasets compared to imbalanced ones	Portugal
15	Fundus image based diabetic retinopathy detection using EfficientNetB3 with squeeze and excitation block [20]	The model uses EfficientNetB3 for diabetic retinopathy detection. It incorporates an attention mechanism for improved classification. Deep features are enhanced using squeeze and excitation blocks. The model is tested on various fundus image datasets	APTOS‐2019 dataset. IDRiD dataset Messidor‐2 dataset	The performance metrics include accuracy, precision, sensitivity, specificity, F1 score, and kappa score. Metrics are derived from a confusion matrix of TP, TN, FP, and FN. The proposed model achieved 88.43% accuracy and 0.88 Kappa score. AUC values indicate model performance in distinguishing classes	The study utilizes EfficientNetB3 for improved accuracy. It incorporates advanced augmentation techniques for better model performance. The classification method is based on contextual information. Limited information on ethical approval for human subjects. The study may lack extensive validation against existing methods	India
16	Image transformers for diabetic retinopathy detection from fundus datasets [21]	Deep learning architectures such as CNN, transformers, and mixer models were used. 18 pretrained models were evaluated for DR detection	APTOS dataset. Messidor dataset. IDRiD‐DR dataset. IDRiD‐AMD dataset. AREDS dataset	F1 score indicates the DR detection capability of the model. Models achieved F1 scores above 86% on APTOS dataset. Swin model had the highest F1 score of 88.7% [2]. Overall F1 scores reached 99.3% for APTOS‐DR detection	Strength: Early disease detection through proposed DR detection framework. Utilizes multiple state‐of‐the‐art models for benchmarking. Comprehensive evaluation on various open‐source datasets. Weakness: Limited to specific retinal disease datasets. Hyperparameter optimization may not generalize across all datasets	India
17	Research on grading detection methods for diabetic retinopathy based on deep learning [22]	The model is based on the vision transformer (ViT) architecture. It integrates local structure characteristics of CNNs using convolution operations	DR images from several public repositories[. Messidor‐2 dataset. APTOS2019 dataset. EyePACS dataset. IDRiD dataset	The model achieved an accuracy of 89.35% across datasets. Precision was reported at 90.12%, indicating strong performance. Detection accuracy reached around 0.88 in validation	The model achieved a high detection accuracy of around 0.88. It provides interpretable justifications for clinical diagnosis. Utilizes publicly available datasets for training and validation. The model structure is based on the innovative vision transformer architecture. Potential weaknesses in generalizability to diverse populations are not addressed. Limited information on the model’s performance in real‐world settings	China
18	Revolutionizing diabetic retinopathy detection using DB‐SCA‐UNet with drop block‐based attention model in deep learning for precise analysis of color retinal images [23]	The proposed model is named Drop Block‐based Spatial‐Channel Attention U‐Net (DB‐SCA‐UNet). The model replaces original U‐Net blocks with channel dropout blocks	DRIVE. STARE. CHASE DB1 Local digital diabetic retinopathy (LDDR)	The model’s performance is evaluated using sensitivity, specificity, accuracy, and F1 score. The proposed model achieved a sensitivity of 0.8781 and an accuracy of 0.9940	Strength: Utilizes advanced deep learning techniques for DR detection. Achieves high sensitivity in retinal vessel segmentation. Reduces workload for eye specialists with automated diagnosis. Weakness: Challenges in microvasculature feature loss at vessel ends. Interference from lesions complicates accurate segmentation	India
19	Segmentation and classification of diabetic retinopathy using ensemble deep neural network [24]	The study employs a cascaded voting ensemble deep neural network model. It integrates multiple algorithmic insights for improved classification	Kaggle dataset. HRID image database. UCI machine learning repository’s dataset	The proposed methodology shows exemplary sensitivity and specificity metrics. It achieves high accuracy, precision, and F1 score in classification. Performance metrics are superior compared to other methods in DR grading	Strength: Utilizes advanced feature extraction techniques for accuracy. Introduces a novel ensemble deep neural network model. Aligns classifications with a standard grading system. Weakness: Image processing methods are not novel. Relies on publicly available datasets, which may limit diversity	India
20	URNet system for recommending referrals for community screening of diabetic retinopathy based on deep learning [25]	URNet employs a GAN‐CNN two‐stage network structure	DDR dataset containing 13,673 fundus images classified into five stages according to the International Clinical Diabetic Retinopathy Severity (ICDRS). A fundus image enhancement dataset containing 1000 sets of clear images and their corresponding low‐quality images	The noise reduction algorithm was evaluated using SSIM and PSNR metrics. Classification performance metrics included precision, recall, F1 score, and accuracy. The ROC curve’s area under the curve (AUC) indicates classification performance	Strength: URNet improves classification accuracy with attention module. Handles abnormal images effectively in community screening. Reduces data labeling burden by bypassing lesion detection. Weakness: Algorithm struggles with complex noise types in images. Limited by varying device impacts on image quality	China
21	Development and analysis of deep learning model based on multiclass classification of retinal image for early detection of diabetic retinopathy [26]	The model uses a convolutional neural network (CNN) architecture. It employs transfer learning with EfficientNet architecture	Kaggle’s Diabetic Retinopathy Detection APTOS 2019 blindness detection dataset	The model achieved a training accuracy of 94.42%. The testing accuracy of the model was 81.81%	The model achieves high accuracy in DR detection. Early detection capability enables timely interventions. Utilizes advanced CNN and EfficientNet architectures. Performance evaluated on an independent test set. Limited to specific retinal image datasets. May require extensive computational resources for training	India
22	A multi‐stage detection of diabetic retinopathy in fundus images using convolutional neural network [27]	The model uses the EfficientNetB3 architecture for classification	Kaggle repository dataset containing 88,702 images, with 35,126 labeled and 53,576 unlabeled images. Kaggle EyePACs dataset. Messidor dataset containing 1200 FDI. IDRiD dataset containing 413 scans	The model’s performance is evaluated using accuracy, specificity, precision, and recall. The model achieved 94.2% accuracy on training data and 96.7% on testing data. The kappa score for the proposed model is 0.874	Strength: High accuracy of 96.7% on testing data. Fully automated system for early disease detection. Utilizes advanced deep learning techniques for image analysis. Weakness: Current techniques are expensive and time‐consuming. The prediction rate is insufficient for a real‐time application	India
23	A new detection model of microaneurysms based on improved FC‐DenseNet [28]	The model is named MAs‐FC‐DenseNet. The model enhances contrast and reduces noise in images	FFA image cohort constructed with collaboration from the Affiliated Eye Hospital of Nanjing Medical University, the first Affiliated Hospital of Soochow University, and Huai’ First People’s Hospital. Dataset containing 1200 FFA images (768 × 868 pixels) from 1200 eyes of DR patients (age range 31–81 years old). Subsets of 960, 120, and 120 FFA images were randomly selected for training, validation, and testing, respectively	Six metrics evaluated MA detection performance: PA, MPA, Pre, Re, F1, and MioU. MAs‐FC‐DenseNet achieved high metric values compared to other models. Metrics included pixel accuracy, mean pixel accuracy, precision, recall, F1 score, and MIoU	MAs‐FC‐DenseNet shows high detection accuracy for microaneurysms. Utilizes advanced preprocessing techniques for improved image quality. Outperforms other models in multiple evaluation metrics. The model may require further feature‐learning enhancements. Missed and false detections still occur in some cases	China
24	AI‐based automatic detection and classification of diabetic retinopathy using U‐Net and Deep learning [29]	The approach uses a two‐stage novel classification system. Independent U‐Net models segment optic disc and blood vessels	Messidor‐2. EyePACS‐1 DiaRetDB0	The model’s performance is evaluated using precision and F1 score Sensitivity, accuracy, specificity, and AUC are also included as metrics. The proposed methodology shows state‐of‐the‐art performance across datasets	Strength: The model achieves high accuracy in DR detection across multiple datasets, utilizing advanced DNN and transfer learning techniques. It employs effective preprocessing and data augmentation methods. Weakness: Dataset imbalance may affect model performance. Limited to specific retinal image datasets	China
25	Automated diabetic retinopathy screening for primary care settings using deep learning [30]	The model is based on deep learning techniques. It utilizes a cloud‐based platform for screening	KG‐set: A dataset containing 88,702 high‐resolution fundus images of people with varying stages of diabetic retinopathy (DR) from EyePACS. MD‐set: A dataset containing 1748 high‐resolution fundus images from the Messidor research program. Messidor‐2: A collection of DR examinations consisting of two macula‐centered eye fundus images per examination	The model achieved 99.21% sensitivity and 97.59% specificity on the KG‐set. AUC for the KG‐set was 0.9992, indicating high performance. On the MD‐set, sensitivity was 97.63% and specificity was 99.49%. Overall sensitivity in primary care was 92.3% with 94.8% specificity	The model utilizes advanced deep learning techniques for DR screening. High sensitivity and specificity were achieved in DR detection models. Ensemble methods improve performance over single‐architecture models. Publicly available datasets enhance reproducibility and validation. Potential patent indicates innovation in screening technology. Limiting to specific datasets may affect generalizability. Further research is needed for clinical feasibility confirmation	USA
26	Automatic detection and segmentation of optic disc using a modified convolution network [31]	The model uses a modified convolutional network for segmentation. VGG16 framework serves as the encoder in the model	Messidor DRIVE DiaRetDB0 DiaRetDB1 CHASE‐DB1 IDRiD STARE	The proposed framework uses intersection‐over‐union, dice coefficient, accuracy, and sensitivity metrics. The algorithm achieved an overall accuracy of 99.44% in segmentation tasks. Dice coefficient and sensitivity are prioritized over accuracy for segmentation evaluation	The accuracy of the study during segmentation is high (99.44%), and the effective detection is made with the help of a modified convolutional network. It uses convolutional long short‐term memory to improve convergence and measure performance by various measures, such as the dice coefficient. It can be restricted to particular databases to test its effectiveness, and it can consume large computational resources to execute it	India
27	Automatic diabetic retinopathy diagnosis using adaptive fine‐tuned convolutional neural network [32]	The model employs a two‐stage transfer learning method. A pretrained convolutional neural network (CNN) is utilized	E‐Ophtha EyePACS Messidor	The system’s performance was evaluated using accuracy, sensitivity, specificity, and AUC	The framework takes advantage of deep learning in screening diabetic retinopathy. It uses a two‐phase transfer learning technique to achieve better precision and minimize the complexity of the model in order to prevent overfitting problems. Achieves state‐of‐the‐art performance on benchmark datasets. The model necessitates large, annotated datasets to perform effectively, and reliance on a subset of datasets could be a constraint to generalizability	Saudi Arabia
28	Automatic diagnosis of diabetic retinopathy with the aid of adaptive average filtering with optimized deep convolutional neural network [33]	The model involves three phases: preprocessing, segmentation, and classification	High‐resolution fundus (HRF) image database. DiaRetDB0. DiaRetDB1_v1. DRIVE database. Kaggle platform	The study analyzed accuracy, sensitivity, specificity, precision, FPR, FNR, NPV, FDR, F1 score, and MCC. The proposed model showed improved performance metrics compared to existing methods	The model applies modified chicken swarm optimization to achieve enhanced accuracy and applies different algorithms to enhance segmentation and classification. The quality of the image can be poor, which can result in inaccurate results. Limited performance metrics were analyzed to validate the model	India
29	A multimodel image enhancement and tailored U‐Net architecture for robust diabetic retinopathy grading [34]	The study proposes a novel deep‐learning framework for DR diagnosis. Classification is performed using CNN, DenseNet‐121, and RefineNet‐U	APTOS 2019 dataset—consists of 3662 annotated retinal fundus images and 1928 unannotated images, categorized into five classes of diabetic retinopathy. DDR (diabetic retinopathy) dataset—comprises 13,673 color fundus images collected from 9598 individuals, with a gender distribution of 48.23% male and 51.77% female. Messidor‐2 dataset—provides a benchmark dataset with high‐quality expert labels for diabetic retinopathy	The proposed model achieved an accuracy of 99.11% and AUC of 98.97%	The study employs a publicly derived anonymized dataset and has better metrics of performance than the state‐of‐the‐art algorithms, a data collection methodology that can effectively extract key characteristics in the classification, and no direct human subjects were used in the study. It is restricted to particular datasets, which can impact their generalizability, and no discussion is made on the potential biases of data selection	India
30	A new approach for detecting fundus lesions using image processing and deep neural network architecture based on YOLO model [35]	The approach utilizes image processing and deep neural networks. It employs data augmentation techniques for training. The YOLOv5 model serves as the architecture basis. Transfer learning is applied using pretrained COCO dataset weights	DR dataset—a public dataset used in the study, containing 757 images labeled in JPEG format with variable sizes. DRiD dataset—another public dataset used for training, adjusting, and testing the proposed approach	The proposed approach achieved an mAP of 0.2630 for IoU limit 0.5. F1 score of 0.3485 was obtained during validation. Precision and recall are key metrics for evaluation. mAP helps evaluate deep learning models for object detection	The research employs high‐image processing methods of detection and is better than the available literature. There is still a problem in identifying very small lesions, with challenges depending on the quality of public datasets	Brazil
31	Cheetah optimized CNN: A bio‐inspired neural network for automated diabetic retinopathy detection [36]	The model is a cheetah optimized convolutional neural network (CO‐CNN). It utilizes a bioinspired classification approach for DR detection	IDRiD dataset—contains a total of 516 images with distinct pathological infections, of which 413 images were used for training	The model achieved an accuracy of 98.64% across all metrics	DR detection accuracy of 98.64, efficiency through CNN optimized with cheetah and improved interpretability by fuzzy logic. The complex grading system requires trained doctors that may lead to decreased performance with complex data	India
32	Deep and densely connected networks for classification of diabetic retinopathy [37]	The model utilizes deep and densely connected networks. It classifies retinal images for diabetic retinopathy stages. The model employs dense blocks with various hyperparameters. It uses threefold cross‐validation to prevent overfitting	Messidor‐2. EyePACS. Kaggle datasets	The study calculates confusion matrix and AUC for performance evaluation. F1 score, precision, sensitivity, and specificity are key metrics used. AUC is computed for each class using ROC curves. Overall accuracy for Messidor‐2 and EyePACS is 0.97 and 0.88, respectively	The model was very accurate in the classification of diabetic retinopathy. Used deep and highly interconnected networks to enhance the performance. It has been tested on huge data such as Messidor‐2 and EyePACS. Patch size and possible labeling errors in the Messidor‐2 dataset: slow training and inference	Korea
33	Deep feature vectors concatenation for eye disease detection using fundus image [38]	The study applied DenseNet121 and Inception‐ResNetV2 as feature extractors	Dataset for Diabetic Retinopathy (DDR) dataset. DiaRetDB1. EyePACS. Messidor. E‐Ophtha. HRF. IDRiD. STARE. Kaggle	DenseNet121 achieved an accuracy of 89.3% with precision, recall, and F1 score of 89%. Inception‐ResNetV2 had an accuracy of 88.8% with similar precision, recall, and F1 score. The proposed model improved performance metrics compared to single models	The research attained a 91% accuracy via a concatenated model. It employed dual CNN models to improve feature extraction. The approach augments diagnostic efficacy for diabetic retinopathy. No study limitations or weaknesses were identified	Indonesia
34	Deep feed forward neural network–based screening system for diabetic retinopathy severity classification using the lion optimization algorithm [39]	The model uses a five‐phase DFNN‐LOA approach. The model is evaluated on the Messidor dataset	Messidor dataset with 1200 instances, including 540 normal 153 mild NPDR 120 moderate NPDR 127 severe NPDR 260 PDR. Publicly accessible datasets for testing the proposed approach	accuracy, sensitivity, specificity, F1 score, PPV, and NPV of 97.6%, 98.4%, 90.7%, 96.5%, 94.6%, and 97.1%, respectively	The model achieves 97.6% accuracy in DR detection, improves sensitivity and specificity in diagnosis, and uses a five‐phase DFNN‐LOA model for efficiency. Lack of optimization algorithms in previous models, and limited to the Messidor dataset for experimental analysis	Germany
35	Deep learning approach for stages of severity classification in diabetic retinopathy using color fundus retinal images [40]	The model uses a deep CNN‐based architecture. Transfer learning is employed to enhance model performance. The model classifies fundus images into severity levels. It was trained on IDRD images for high accuracy	IDRiD dataset. Messidor‐1 diabetic retinopathy. APTOS 2019 blindness detection. DR2 database. Messidor database	Performance metrics include accuracy, precision, recall, F‐score, and AUC. The ROC curve assesses model efficiency through sensitivity and specificity. LR classifier outperformed others in precision, accuracy, and F1 score	The model is quite accurate in the classification of diabetic retinopathy, makes use of the CNN to find lesions effectively, and uses data preprocessing to improve the results. Training models massive amounts of data and image interpretation is still a problem	India
36	Deep learning‐based diabetic retinopathy recognition and grading: Challenges, gaps, and an improved approach—A survey [41]	The model utilizes an ensemble EfficientNet for classification. It incorporates IoMT for diabetic retinopathy diagnosis. The approach enhances classification through multitask learning. It employs a stacked ensemble‐based classification model	EyePACS, STARE, APTOS, DRIVE, HEIMED, DIARETDB, ROC, E‐Ophtha, Messidor, RFMiD, DDR, OCTID, Duke OCT, UCSD	STMFNet achieved 97.45% accuracy on the EyePACS dataset. The model reached 96.82% accuracy on the Messidor dataset. APTOS dataset performance was 96.51% accuracy. IDRiD dataset showed 95.90% accuracy. Precision on EyePACS was 98.90%. F1 score for Messidor was 96.95%. APTOS F1 score was 96.65%	It employs the state of the art deep learning methods in DR detection, and it is sensitive to subtle retinal pathologies. There is, however, little discussion on actual clinical application, and it may be inefficient in terms of required computational resources	South Korea
37	Deep learning frameworks for diabetic retinopathy detection with smartphone‐based retinal imaging systems [42]	The study developed an automatic DR detection model. It utilized deep learning with the ResNet‐50 network. Transfer learning was applied using pretrained networks: AlexNet, GoogLeNet, and ResNet‐50	EyePACS, Messidor, Messidor‐2, Indian Diabetic Retinopathy Image Dataset (IDRiD), and University of Auckland Diabetic Retinopathy (UoA‐DR)	ResNet‐50 achieved 98.6% accuracy, 98.2% sensitivity, and 99.1% specificity. ROC curves indicated best performance for original retina images. EER values ranged from 0.026 to 0.528 for various thresholds. High sensitivity for DR was 93.3% with 90% specificity	The classification accuracy of 98.6% was as high as the sensitivity of 98.2% and specificity of 99.1%. It involves the use of transfer learning to better detect DR, and explores the impact of field of view on the accuracy of detection. It possesses only a few training images as compared to the existing studies	United States
38	Deep red lesion classification for early screening of diabetic retinopathy [43]	A deep CNN model is utilized for DR diagnosis. The ResNet‐50 architecture is modified to create DR‐ResNet‐50	E‐Ophtha_MA. IDRiD. Four lesion‐level annotated datasets. Two image‐level annotated datasets	The model’s performance was evaluated using accuracy, precision, and sensitivity metrics. AUC, F1 score, and Matthews correlation coefficient were also computed. The highest sensitivity and specificity scores were 0.9851 and 0.991, respectively The false‐positive rate was reported as 0.0029	Very high sensitivity and low false‐positive rate and were obtained, strong performance on five public datasets, and the method is much simpler than classical methods. It is difficult to train deep models with small data, and the problem of potential degradation of performance and overfitting is observed	Pakistan
39	Detection of fundus lesions through a convolutional neural network in patients with diabetic retinopathy [44]	The model is based on YOLOv4 architecture. It utilizes pretrained convolutional neural networks for detection	DDR fundus image dataset—contains 757 images with annotations of the fundus in JPEG format	The model achieved an mAP of 11.13% and mIoU of 13.98%. AP values for lesions were 5.72% (EX), 21.62% (SE), 1.52% (MA), 15.66% (HE). The lowest loss function value was 31.26%	The model makes use of pretrained convolutional neural networks in terms of accuracy. It has better results than related work. mAP and mIoU values are rather low	Brazil
40	Development and validation of a deep learning model for early detection and screening of diabetic retinopathy [45]	The model utilizes zero‐shot learning for DR detection. The model employs Dice loss for improved segmentation performance	MIMIC‐CXR dataset CBIS‐DDSM dataset MURA‐1.1 dataset DRIVE dataset ODIR‐5k dataset FIRE dataset STARE dataset CHASE‐DB1 dataset IDRiD dataset	Sensitivity and specificity exceeded 85% for most datasets. F1 score and AUROC remained above 80% across datasets. Average precision (AP) assesses precision–recall trade‐off at the image level	The model utilizes zero‐shot learning to minimize labeled data reliance, enhancing detection accuracy for early stage diabetic retinopathy lesions, and employs advanced techniques like contrastive learning for feature capture. Limited by the availability of public datasets and potential privacy concerns with medical data handling	China
41	Diabetic retinopathy detection using genetic algorithm‐based CNN features and error correction output code SVM framework classification model [46]	The model uses a deep neural network approach. Five CNN architectures extract features: AlexNet, NASNet‐Large, VGG‐19, Inception V3, ShuffleNet	Kaggle dataset (8 GB labeled dataset). Custom dataset (containing retina fundus images). Enhanced custom dataset (developed through data augmentation)	Performance metrics include accuracy, precision, recall, and F1 score. The model achieved 97.9% accuracy on the Kaggle dataset. The custom dataset yielded 94.76% accuracy. The enhanced custom dataset achieved 96.4% accuracy	Better results are obtained with dense 169 model, where high accuracy on validation set is obtained with GPU, and the strategy facilitates clinical decisions. It is constrained by the small size of datasets and the requirement to use additional patient data, The difficulty in identifying the .degree of diabetic retinopathy severity	Pakistan
42	Diabetic retinopathy detection using red lesion localization and convolutional neural networks [47]	The study designed a lesion localization model using a patch‐based approach. The model employs two convolutional neural network models for training patch selection	Standard Diabetic Retinopathy Database, Calibration Level 0 (DiaRetDB0). Standard Diabetic Retinopathy Database, Calibration Level 1 (DiaRetDB1). Kaggle retinal dataset. Messidor. Messidor, Indian Diabetic Retinopathy Image Dataset (IDRiD). DDR dataset	The model achieved an AUC of 0.912 for DR screening. Sensitivity reached 0.940, indicating high detection capability	The paper employs the fully automatic CNN technique of lesion localization. It offers such performance measures as AUC, sensitivity, and specificity. Bootstrap method increases reliability of recall, precision, and F1 score. The experiment is restricted to test certain diabetic retinopathy databases only and cannot be applied to all retinal image databases	Brazil
43	Diabetic retinopathy detection using smartphone fundoscopy: advancing healthcare equity through intelligent edge computing [48]	The model uses EfficientNetB0 architecture for diabetic retinopathy detection. It is pretrained on the ImageNet dataset for feature extraction	APTOS 2019 dataset—comprises retinal pictures exhibiting different phases of diabetic retinopathy	The EfficientNetB0 model achieved a total accuracy of 97%. The model’s performance was superior to other preexisting models. The F1 score metric was used for evaluation	The model is based on making use of smartphones to conduct diabetic retinopathy screening, EfficientNetB0 model has high accuracy in detection, cost‐effective in the underserved areas and enables communities to empower themselves to conduct self‐screening. There is restricted availability of trained specialists in the remote areas and inconsistency of the quality of screening because of the equipment constraints	India
44	Diagnosis of retinal disorders from optical coherence tomography images using CNN [49]	A convolution neural network (CNN) model is proposed for classification. The model classifies three retinal disorders: CNV, DMD, and DME. A novel CNN model with less complexity is introduced	A dataset of 12,000 images was used, with 3000 images in each category: choroidal neovascularization (CNV), Drusen macular degeneration (DMD), diabetic macular edema (DME), and normal images. The study focused on optical coherence tomography (OCT) images of different retinal disorders	The proposed model achieved 97.01% accuracy and 93.43% sensitivity. Specificity of the model was reported at 98.07%. ROC rates for classes ranged from 96% to 99%	The model has demonstrated high accuracy, achieving a classification rate of 97.01%. It is utilized in the screening of retinal disorders in large‐scale and employs the advanced CNN architecture, thus achieving better performance. The model is perplexed when it tries to predict certain images and thus makes errors that are only restricted to certain retinal disorders (CNV, DMD, DME)	India
45	Early detection of diabetic retinopathy using PCA‐Firefly based deep learning model [50]	The model uses PCA for feature extraction. Firefly algorithm is applied for dimensionality reduction. A deep neural network is utilized for classification. The model outperforms other popular machine learning models	Diabetic retinopathy dataset with 1151 instances and 20 attributes, collected from the UCI machine learning repository	Accuracy measures correct predictions against actual values during testing. Sensitivity indicates true positives identified by the classifier. Specificity reflects true negatives correctly identified by the classifier. F1 measure represents the harmonic mean of precision and recall. Recall determines the percentage of positive instances identified as positive. The proposed model outperforms others in accuracy, precision, recall, sensitivity, and specificity	The model makes use of PCA to reduce dimensionality effectively. Applies Firefly algorithm to enhance performance on the classification. It has a high level of accuracy, precision, and recall. The model is restricted to a particular dataset of diabetic retinopathy; it might not be generalized to other datasets and conditions	India
46	Efficient diabetic retinopathy detection using deep learning approaches and Raspberry Pi 4 [51]	The study uses DenseNet for diabetic retinopathy detection. DenseNet captures complex features from diverse datasets. The model is integrated into a Raspberry Pi 4	DRIVE Messidor Kaggle diabetic retinopathy detection (Kaggle‐DRD) E‐Ophtha APTOS 2019 blindness detection IDRiD Messidor‐2	The proposed approach achieved a classification accuracy of 88% in DR detection. Performance assessment utilized a confusion matrix for accurate predictions	The model had high classification rate of 88 percent. it employs the DenseNet feature extractor, which is able to extract complex features, and it is integrated in a Raspberry Pi that can be readily accessed. Weak debate on issues of model generalization and missing points in the available literature are not adequately addressed	India
47	Efficient microaneurysm segmentation in retinal images via a lightweight Attention U‐Net for early DR diagnosis [52]	The model utilizes a lightweight Attention U‐Net architecture. It incorporates an optimized preprocessing pipeline for image enhancement	IDRiD dataset—comprises 516 retinal fundus images, with 81 images specifically used for MA segmentation. E‐Ophtha dataset—includes a subset of 148 images with annotated MAs	Evaluation metrics include sensitivity, specificity, accuracy, precision, and dice coefficient. The model achieved a sensitivity of 0.81 and specificity of 0.99 on IDRiD [2]. On the E‐Ophtha dataset, sensitivity was 0.59 and specificity was 0.99	Introduces a new preprocessing system to work with image improvement. It uses a fast attention U‐Net to conduct efficient segmentation and attains high specificity and decent sensitivity in assessments. The performance of sensitivity is considerably different in various datasets. There are still problems in identifying small variable size microaneurysms	China
48	Efficient transformer architectures for diabetic retinopathy classification from fundus images: DR‐MobileViT, DR‐EfficientFormer, and DR‐SwinTiny [53]	Three lightweight transformer‐based models were developed for DR classification [1] DR‐MobileViT combines convolutional and transformer layers for feature extraction. DR‐EfficientFormer uses patch embedding with scaling dimensions for classification. DR‐SwinTiny employs shifted window attention and patch merging layers	APTOS 2019 Blindness Detection dataset (3662 fundus images). Messidor‐2 dataset (1748 high‐resolution fundus images	The models achieved QWK scores up to 0.89. AUC scores reached up to 0.95. Evaluation metrics included sensitivity and specificity. Performance metrics averaged over five runs for robustness	Presents three light transformer models of DR classification, performing well on diagnostic accuracy at lower computational levels. The models have a rapid inference time of 10–15 ms per image and are suitable to resource constrained environments. The testing is restricted to various datasets, ablation studies are not comprehensive, visual interpretability studies are omitted, and multimoded data are not employed to increase the accuracy level	Algeria
49	Ensemble EfficientNet: a novel technique for identification, classification, and prediction of diabetic retinopathy [54]	The study introduces ensemble EfficientNet for diabetic retinopathy detection. It utilizes the Kaggle DR dataset for model training	Kaggle DR dataset. IDRiD. Ocular recognition. Retinal dataset. H.R.F. DRIVE	The model achieved 95% accuracy and 97% recall in D.R. detection. Evaluation metrics include accuracy, precision, and recall. The confusion matrix provides insights into model performance	The model has 95% accuracy in the detection of diabetic retinopathy and a high recall rate of 97 percent in detection cases of DR, and it makes use of ensemble learning as a means of improving model robustness. It needs additional investigation using various datasets to be consistent	India
50	Ensemble of multi‐stage deep convolutional neural networks for automated grading of diabetic retinopathy using image patches [55]	The paper proposes an ensemble of deep CNN models for DR detection. Pretrained models InceptionV3 and Xception are utilized	Diabetic retinopathy database (DIARETDB). Structured analysis of retina (STARE). E‐Ophtha. Retinopathy online challenge (ROC). Kaggle database. BH dataset. LFH dataset (captured using Zeiss FF 450 PLUS IR fundus camera)	The overall classification accuracy achieved is 96.2% with MPDCNN. Individual accuracies for classes are 97%, 95%, 96%, and 97%. Performance measures include TPR, TNR, PPV, QI, F score, and MCR	The model demonstrated a classification accuracy of 96.2%. It employs a combination of multistage deep CNN models and an automated grading system for diabetic retinopathy. Manual screening continues to be labor‐intensive and is restricted to certain retinal lesion identification	India
51	Hard exudate detection based on deep model learned information and multi‐feature joint representation for diabetic retinopathy screening [56]	The method uses a deep convolutional neural network (DCNN) for HE detection. It combines deep features with hand‐crafted features for improved accuracy	Standard Diabetic Retinopathy Database Calibration Level 1 (DI‐ARETDB1)—consists of 89 retinal images with rough exudate annotations. Hamilton Eye Institute Macular Edema Dataset (HEI‐MED)—consists of 169 color fundus images, including 115 healthy images and 54 images with hard exudates (HEs). E‐Ophtha—comprises 82 fundus images with manual segmentations of HEs, including 35 healthy and 47 diagnosed with HEs	Sensitivity and PPV are key metrics for HE segmentation performance. F score is significant, indicating high sensitivity and PPV. AUC values characterize classifier performance; closer to 1 is better. MRBS outperforms OMRBS with sensitivity of 0.9113 and PPV of 0.9418. FSF improves performance with AUC values of 0.9647 and 0.9653	The technique achieves state‐of‐the‐art results in DR screening and HE detection by combining deep features with conventional HCFs. High Se with low PPV or vice versa are among the method’s drawbacks, and overall performance might not be optimal	China
52	Hybrid quantum‐classical deep learning framework for balanced multiclass diabetic retinopathy classification [57]	The model combines a ResNet‐based feature extractor and quantum processing layer	APTOS 2019 Blindness Detection dataset. Indian Diabetic Retinopathy Image Dataset (IDRiD). EyePACS. Messidor	The model achieves a balanced accuracy of 80.96% on the APTOS 2019 dataset	The model evaluates several metrics, including precision, recall, and F1 score, and uses deep learning to accurately detect diabetic retinopathy. It also uses an enhanced activation function for better performance. There are only five classes for diabetic retinopathy, and dropout rates may cause overfitting	UAE
53	Improved and robust deep learning agent for preliminary detection of diabetic retinopathy using public datasets [58]	The study utilized Inception (v3) and InceptionResNet (v2) models for classification. Models were trained on the Eye‐PACS dataset for diabetic retinopathy detection	EyePACS dataset (56,839 fundus images for training). EyePACS test set (14,210 images). Messidor‐2 (1748 images). Messidor‐1 (1200 images)	The model achieved AUC of 0.92 on Messidor‐2 dataset. Sensitivity and specificity for Messidor‐1 are 88.84% and 89.92%, respectively	The model uses cutting‐edge CNN models for detection and tackles issues in automated medical image detection, achieving high AUC scores on benchmark datasets. Due to low awareness, DR is highly prevalent and has limited deployment in actual clinical settings	India
54	Improved automatic grading of diabetic retinopathy using deep learning and principal component analysis [59]	A novel multilevel classification approach was proposed for diabetic retinopathy detection. The approach combines classical CNN with PCA analysis for better classification	Public DR dataset from the Kaggle competition, containing five levels of DR. Additional dataset obtained from a university hospital for independent testing. Local dataset with around 100 color images divided into five classes	The study achieved 85% accuracy in DR classification. Sensitivity was reported at 89%. Specificity reached 96% in the evaluation	On benchmark datasets, the model’s AUC scores were high. Overcomes obstacles in automated medical image detection by using cutting‐edge CNN models for detection. Due to low awareness, DR is highly prevalent and has limited deployment in actual clinical settings	Egypt
55	Interpretable deep learning for diabetic retinopathy: A comparative study of CNN, ViT, and hybrid architectures [60]	The study evaluates CNNs, ViTs, and hybrid models for DR classification. CNNs include ResNet‐50 and EfficientNet‐B0	EyePACS dataset. APTOS‐2019 dataset	The primary metric used is quadratic weighted kappa (QWK) Models show positive correlation between QWK and other metrics. Hybrid models outperform standalone CNNs and ViTs in performance	Obtains high scores on benchmark datasets, uses state‐of‐the‐art CNN models to detect and also confronts the issue of automated medical image detection. It is a reduced implementation in actual clinical situations and there is a high rate of DR as a result of inadequate awareness	Germany
56	Investigation for diabetic retinopathy detection using a pseudolearning classifier [61]	The model employs a hybrid deep learning architecture. EfficientNet‐L2 CNN is used for feature extraction and segmentation	EyePACS Messidor Clinical datasets	The model achieved 96.5% accuracy and 96.3% F1 score. Precision and recall exceeded 96% across all diabetic retinopathy stages. Pseudolabeling improved robustness and training efficiency	The research proves the high accuracy of DR detection of 96.5, It is efficient to reduce false negatives and positives in clinical diagnosis. The hybrid model takes advantage of the labeled and unlabeled data to be robust. Future research will seek to test in diverse populations and incorporate other modalities. Minimal discussion of semisupervised methods of large‐scale DR detection	India
57	Microaneurysm detection in fundus images based on a novel end‐to‐end convolutional neural network [62]	A novel one‐stage encoder–decoder network is proposed for detection	E‐Ophtha‐MA dataset. Retinopathy Online Challenge dataset. ROC dataset	The proposed model uses free‐response receiver operating characteristic (FROC) for evaluation. Sensitivity and false positive rates are key metrics in the evaluation. The model achieves state‐of‐the‐art sensitivities at various false‐positive indices Model achieves high performance with only 1.678 million parameters	The model is easy and efficient. It ensures performance with shorter test time and has a similar performance to state‐of‐the‐art techniques. Complex backgrounds make the automatic detection difficult. The context does not give much information on weaknesses	China
58	Mobile‐based deep learning system for early detection of diabetic retinopathy [63]	The study employs a deep convolutional neural network (DCNN) model. It utilizes transfer learning with DenseNet‐121 architecture. The model is integrated into a mobile application for real‐time use	APTOS EyePACS Private dataset collected from patients diagnosed with varying stages of diabetic retinopathy (DR) using the oDocs Nun IR camera	Performance metrics include PSNR, SSIM, and RCEF for image enhancement evaluation	MobileNet‐v2 and Inception‐v3 have reduced inference time due to their lighter architecture, and DenseNet‐121 is capable of learning complex retinal features. The model was highly accurate on several datasets, and the system is compatible with the mobile platform, providing real‐time diagnostics. The next step in the work is the better efficiency of models and the real‐life validation	Morocco
59	Optimizing diabetic retinopathy detection with electric fish algorithm and bilinear convolutional networks [64]	The model employs a three‐phase methodology for DR detection	APTOS 2019 Blindness Detection Database, which includes 3662 retinal images taken in different lighting conditions	The study uses F1 score, accuracy, and precision as metrics. MAPCRCI‐DMPLC 22 achieved 0.89 accuracy and 0.86 F1 score ISVM‐RBF showed 0.88 accuracy and 0.87 F1 score. MuR‐CAN achieved 0.90 accuracy and 0.89 F1 score. BCAN demonstrated 0.93 accuracy and 0.94 F1 score	The research proves to be better in high accuracy and precision. It uses highly developed machine learning in the early detection of DR. The analysis of the model indicates that it can perform well on a balanced dataset. Lack of manual diagnosis limits the use of manual diagnosis. The research proposes the broader usage of AI in eye diseases	India
60	Progress of artificial intelligence in diabetic retinopathy screening [65]	The model approach involves collecting data for a DR database. It utilizes CNN systems for DR screening	STructured Analysis of the Retina (STARE)—130 images at level 0 and 89 images at level AusDiab study—includes images of MAs and 233 healthy images. Singapore Integrated Diabetic Retinopathy Program (SiDRP)—over 110,000 screens from diabetic patients. IDRiD—dataset from India. APTOS—dataset from India. LabelMe—dataset from China	Not applicable	High diagnostic AUC of 0.97 was obtained in the study. The AI system had a specificity of 90.7. Image variability and the visibility of lesions were enhanced by data augmentation. A big external test set was used in the study. There are still matters of potential problems in the use of AI in the screening of DRs. The research can be problematic in the real‐life application	China
61	Suitability classification of retinal fundus images for diabetic retinopathy using deep learning [66]	A lightweight convolutional neural network is proposed for binary classification. YOLOv5 model is used for object detection in retinal images. DenseNET‐based CNN modules improve performance and parameter efficiency	Kaggle diabetic retinopathy detection provided by EyePACS (IDRiD) (Messidor ‐2) (APTOS) APTOS2019 (DRIVE) (DRIMDB	The average accuracy is computed during training and validation. Recall or sensitivity and specificity are calculated for evaluation. The area under the curve (AUC) is a performance metric. The proposed model achieves 98.6% AUC and 97.66% sensitivity	The sensitivity and specificity of DR detection are high. The CNN lightweight model makes the computational costs less expensive. Semiautomated workflow improves image classification. Reliance on the quality of input retinal images. Quality assessment is to be done by humans	MexicoIm

## Appendix B

**Table A2:** Thirteen included studies with a machine learning approach.

	Article name and citation	Model approach	Datasets	Performance metrics	Strengths and weaknesses of the study	Study location
1	Microaneurysms detection in color fundus images using machine learning based on directional local contrast [68]	The study proposes a microaneurysms detection method using machine learning. K‐nearest neighbor (KNN) is a nonparametric supervised learning method used. Support vector machine (SVM) is employed for data classification	E‐Ophtha MA database. DiaRetDB1 database	The area under the ROC curve was 0.87 and 0.86 for two databases. The free‐response ROC score was 0.374 and 0.210 for the databases	The experiment was successful with 95.4% accuracy of BV segmentation, and the directional local contrast feature enhanced the MA detection. Using the method, less computational time was required. CNN methods have a drawback of requiring larger training time; physiological factors influencing MA detection have not been taken into account	China
2	Interpretable transfer learning framework for diabetic retinopathy stage classification [69]	The approach utilizes transfer learning for diabetic retinopathy classification. It employs various deep learning architectures for model evaluation. Models include ResNet‐50, InceptionV3, and MobileNetV2	APTOS 2019 Blindness Detection dataset, obtained from Kaggle, consisting of 3662 training images and 1928 testing images. Diabetic retinopathy, Messidor, EyePac, preprocessed datasets, also obtained from Kaggle	Models evaluated include accuracy, precision, recall, and F1 score. VGG16 achieved 99% accuracy for “No DR” class. InceptionV3 recorded 83% accuracy on Dataset A. ResNet‐50 had the highest accuracy on Dataset A at 86%.EfficientNetB0 exhibited the weakest performance with 63% accuracy on the dataset	The model uses recent state‐of‐the‐art CNN models in classification. It has SHAP analysis to understand model interpretability and measures various datasets to have a holistic performance evaluation. The model is not very successful in separating intermediate and developed DR stages, and the obscurity of deep learning models is an ethical issue	Tunisia
3	Hybrid multikernel SVM algorithm for detection of microaneurysm in color fundus images [70]	The study develops an automatic screening system for DR detection. A hybrid multikernel support vector machine classifier is proposed. The model combines shape and texture features for improved performance	DiaRetDB. Messidor. Retinopathy Online Challenge. AGAR300	The FROC curve is used for per lesion evaluation in medical imaging. The proposed model achieved the best FROC scores across datasets	This research offers a combination of shape and texture features to enhance detection efficiency; the number of hand‐crafted features is minimized and, therefore, the method is less computationally intensive. The proposed method is superior to the existing techniques in several metrics. There is misclassification among the classes because of the nature of lesions, and the lack of uniform distribution of classes in SVM is a noted drawback	India
4	Diabetic retinopathy prediction by ensemble learning based on biochemical and physical data [71]	The study proposed the XGB‐stacking model combining XGBoost and stacking methods A SEL‐stacking method was used to optimize learner combinations	A structured dataset of diabetic retinopathy provided by the China National Clinical Medical Science Data Center, which includes numerical and qualitative variables, but no image data. The processed experimental data include a total of 2990 samples, with 68 features and 1 label indicating the presence of diabetic retinopathy	Classification accuracy measures correctly classified samples to total samples. Feature dimensionality reduction indicates unused features versus original features. Only accuracy was used for model fusion evaluation. XGBIBS improved prediction accuracy and feature reduction rate	Good forecasting of diabetic retinopathy was made possible. It was better in accuracy by XGBIBS feature selection and employs ensemble learning to increase model performance. The amount of limited data can influence the generalizability of the model and lead to overfitting because of small size of the sample	China
5	Diabetic retinopathy grading by a source‐transfer learning approach [72]	The model employs source‐free transfer learning (SFTL) for DR detection. It utilizes unannotated retinal images for training. Two major modules are proposed: target generation and collaborative consistency	EyePACS dataset APTOS 2019 dataset	The SFTL model achieved an accuracy of 91.2%. Sensitivity was reported at 0.951. Specificity was noted as 0.858. Evaluation metrics included accuracy, sensitivity, and specificity	The model makes use of the source‐free transfer learning in screening of DR. It also improves performance with no annotated retinal images and classifier performance via cotraining. is only tested on particular datasets, such as the EyePACS and APTOS, and possibly needs additional validation on a wider range of data	China
6	Automatic screening of diabetic retinopathy using fundus images and machine learning algorithms [73]	The model used two machine learning methods: support vector machine (SVM) and deep neural network (DNN)	Kaggle, DDR, Zenodo, and Mendeley	The proposed SVM model had a mean area under the receiver operating characteristic curve (AUC) of 97.11%, whereas the DNN model had a mean AUC of 99.15%	SVM and DNN models were highly accurate. The current diagnosis cannot be made with the help of data that is publicly available because of its size, and it needs to be visualized manually (consuming time)	United Arab Emirates
7	Combining transfer learning with retinal lesion features for accurate detection of diabetic retinopathy [74]	The model uses transfer learning with retinal lesion features. ResNet‐50 TL model extracts features for classification	Ophtha dataset Retinal lesions dataset APTOS 2019 Blindness Detection Kaggle competition training dataset Testing dataset of 162 color fundus images collected from three publicly available sources	U‐Net model performance is evaluated using accuracy, recall, precision, F1 score, and IoU	The model applies transfer learning for better feature extraction. It scores 90 percent in detecting diabetic retinopathy and uses lesion features to represent them more effectively. Only convolutional models (VGG‐16, ResNet‐50) are available, and not all retinal image datasets can be generalized to	Italy
8	Transfer learning based robust automatic detection system for diabetic retinopathy grading [75]	The model utilizes transfer learning for diabetic retinopathy detection. It employs deep neural networks for feature extraction from fundus images. An ensemble of CNNs and SVM classifier is used for grading	Indian Diabetic Retinopathy Image Dataset (IDRiD)—454 images with NPDR severities. Messidor dataset—1200 fundus images with various DR severity grades	The study evaluates metrics such as PPV, NPV, sensitivity, and specificity [1] The proposed model achieved 90.51% accuracy using PFTL method	The classification accuracy of 90.51 was high. This model is based on the transfer learning to achieve better feature extraction and ensemble method increases the performance of the model to a higher level. Restricted to certain datasets to be validated, may take a long time to train	India
9	A deep transfer learning approach for identification of diabetic retinopathy using data augmentation [76]	A transfer learning model was proposed for diabetic retinopathy detection. Utilized ResNet‐50 architecture for classification tasks.	Kaggle dataset Messidor dataset APTOS‐Blindness dataset	The model achieved 91% accuracy using synthetic data. Without synthetic data, the accuracy was 86%. Overall accuracy improved by approximately 11% with synthetic images	This model increased classification accuracy by 11 percent using synthetic images. The overfitting was avoided with the help of data augmentation. A big collection of 13,000 pictures was used in the study. Manual diagnosis is susceptible to mistakes, thus necessitating the need to automate, and there were unbalanced distribution among the models, which also influenced model training	India
10	Implementation of artificial intelligence–based diabetic retinopathy screening in a tertiary care hospital in Quebec: Prospective validation study [77]	The model used is CARA, a semiautomated AI system [1] CARA employs traditional machine learning for feature extraction	Not address	CARA’s sensitivity for referable disease was 87.5% (95% CI 71.9–95.0). CARA’s specificity for referable disease was 66.2% (95% CI 54.3–76.3). Sensitivity for retinopathy detection was 88.2% (95% CI 76.6–94.5). Specificity for retinopathy detection was 71.4% (95% CI 63.7–78.1). DME detection sensitivity was 100% (95% CI 64.6–100) [3] DME detection specificity was 81.9% (95% CI 75.6–86.8)	Superior sensitivity in diabetic macular edema and validation of real‐world performance in a clinical environment. Moderate specificity to referable disease and small sample of 115 patients	Canada
11	Diabetic retinopathy detection via exudates and hemorrhages segmentation using iterative NICK thresholding, watershed, and Chi2 feature ranking [78]	K‐means clustering algorithm used for image segmentation. Two streams of a faster region‐based convolutional neural network employed. VGG‐16 model utilized for ROI pooling, regression, and classification. Combination of SVM and GMM used for classification	A larger dataset from a traditional ophthalmoscope. Two fundus datasets, each containing both normal and diabetic retinopathy (DR) fundus images	INRG‐WS achieved the highest F‐measure of 64.76% for hemorrhage segmentation. INRG‐WS‐HSV had the highest accuracy of 88.14% for exudate detection. INRG‐HSV achieved 90.27% accuracy, with a false‐negative rate of 9.39%. Overall accuracy of 89.89% with a false‐negative rate of 3.66%	The model involves the use of a bigger dataset to enhance accuracy and complex hemorrhages and exudate segmentation techniques. It is limited to certain types of diabetic retinopathy and can be very computationally intensive	Thailand
12	Contrastive self‐supervised learning for diabetic retinopathy early detection [79]	The model uses a two‐stage approach: pretext and downstream tasks. Contrastive learning pretrains the encoder with unlabeled data. The encoder is fine‐tuned with a small labeled dataset	Kaggle competition dataset provided by EyePACS, containing 35,126 retinal images from 17,563 patients, with labels indicating the severity of diabetic retinopathy (DR). Subsets of the original Kaggle dataset for the Pretext task and downstream task, divided with a ratio of 9:1 in each class. Unlabeled training set, with 1%, 10%, or 20% of it relabeled for the downstream task	The proposed model’s performance is evaluated using accuracy. Accuracy is defined as TP + TN/(TP + TN + FP + FN). The model addresses data insufficiency in diabetic retinopathy detection	The model applies unlabeled learning that is self‐supervised. It gets around the issue of a lack of training data and achieves better results in DR classification. It is constrained to small, annotated training data for retraining and depends on the retinal image quality of unlabeled images	China
13	Modified deep inductive transfer learning diagnostic systems for diabetic retinopathy severity levels classification [80]	The approach utilizes deep inductive transfer learning (DITL)	IDRiD dataset (Indian Diabetic Retinopathy Image Dataset). APTOS (Asia‐Pacific Tele‐Ophthalmology Society) dataset. DiaRetDB1 dataset. Messidor‐1 dataset Messidor‐2 dataset	The model’s accuracy is 0.9840, indicating effective classification Precision for Stage 0 is 0.97, Stage 1 is 0.99, and Stage 2 is 0.98 Recall for Stage 0 is 0.99, Stage 1 is 1, and Stage 2 is 0.94. AUC is 0.9979, demonstrating strong class differentiation. F1 scores are 0.98 for stage0, 1 for Stage 1, and 0.96 for s Stage 2	The research applies deep inductive transfer learning models. It is very accurate in multiclass DR severity classification. The model performs well on class imbalance using upsampling. The dataset comprises the extensive scope of DR phases. There is a significant improvement in the Xception model performance. There is not much information on possible overfitting. It may be necessary to provide more data in the research to make it more universal	India

## Appendix C

**Table A3:** Twenty included studies with other approaches.

	Article name and citation	Model approach	Datasets	Performance metrics	Strengths and weaknesses of the study	Study location
1	Diabetic retinopathy detection based on mobile maxout network and Weber local descriptor feature selection using retinal fundus image [81]	A hybrid network structure for DR detection utilizing retinal fundus image named Rmaxout network (MM‐Net). MM‐Net is merged with the merging of MobileNet and deep maxout network (DMN)	Indian DR Image Dataset	MM‐Net achieved accuracy of 89.2% in DR detection. Sensitivity and specificity were 90.5% and 92.0%, respectively	MM‐Net demonstrated high accuracy in DR detection; the generalization ability of WFDLN was observed despite the difference in class sizes, and the stability and reliability of MGS‐ROA‐DBN were observed in the feature selection. Huge imbalance and overfitting problems of TL‐CNN and missing feature extraction methods of NIMEQ‐SACNet influence performance	India
2	Identification of the optimal model for the prediction of diabetic retinopathy in Chinese rural population: Handan Eye Study [82]	The study established five prediction models for diabetic retinopathy (DR). Models included MLR, C&RT, SVM, RF, and GBM. MLR was identified as the optimal model for DR prediction	Data from the Handan Eye Study (HES) involving 4752 participants was used to build the diabetic retinopathy (DR) prediction model. Private patient data from HES, which are not publicly available, were analyzed in the study	The MLR model’s AUROC was 0.936, with sensitivity 0.914 and specificity 0.898. The GBM model’s AUROC was 0.952, with sensitivity 0.943 and specificity 0.881. The C&RT model’s AUROC was 0.682, with sensitivity 0.371 and specificity 0.992. The study assessed models based on AUROC, sensitivity, specificity, and accuracy	The model makes use of a Handan Eye Study with a high sample size. It also compares the performance of traditional and machine learning algorithms in prediction. and the MLR model demonstrates the outstanding prediction performance in DR. Dataset is personal patient data and not publicly accessible and generalizability is restricted to Chinese rural population	China
3	Assessing the diagnostic capabilities of ChatGPT‐4 Omni in grading diabetic retinopathy Fundoscopy using color fundus photographs [83]	ChatGPT simulates an ophthalmologist for diabetic retinopathy analysis. The study utilized the EyePACS dataset for evaluation	EyePACS DR detection competition dataset from Kaggle, which includes 2500 color fundus photographs representing no DR, mild DR, moderate DR, severe DR, and proliferative DR	ChatGPT‐4o achieved an accuracy of 0.756 for proliferative DR. Overall accuracy ranged from 70% to 80% for various DR stages	The model assesses the low‐cost AI in diabetic retinopathy screening. It employs a massive amount of 2500 color fundus photographs and is prospective to identify serious cases of diabetic retinopathy. Difficulties in classifying diabetic retinopathy at the initial stages. Reduced sensitivity and specificity than approved systems by the FDA and Misclassifications between adjacent levels of diabetic retinopathy	USA
4	Automated detection of hard exudates in retinal fundus images for diabetic retinopathy screening using textural‐based radon transform and morphology reconstruction [84]	The model integrates textural‐based Radon transform and morphology reconstruction	DiaRetDB1—A publicly available dataset consisting of 89 high‐resolution CFP images, covering the full spectrum of diabetic retinopathy (DR) stages, with images annotated by expert ophthalmologists. MUMS‐DB—a local dataset comprising 32 high‐resolution CFP images focusing on retinal abnormalities in DR patients, also annotated by expert ophthalmologists	The algorithm achieved 92% sensitivity and 100% specificity in pixel‐based classification. In lesion‐based classification, it reached 100% sensitivity and specificity on MUMS‐DB	The model has a high sensitivity (92%) and specificity [53] in detection. Intense way and no cropping of pictures required. It integrates clinical experience with computer methods to make an improved diagnosis. The data is quite different in performance, and difficulties in creating all‐purpose robust algorithms are observed	Iran
5	Use of serum long noncoding RNA expression panel as a marker for diabetic retinopathy [85]	The study used lncRNA expression profiles to detect diabetic retinopathy (DR)	Not addressed in the paper	The lncRNA model had 81% sensitivity and 63% specificity. The demographic data‐only model had 94.7% specificity and 62.5% sensitivity	The research has a varied population of patients. It makes use of HbA1c data to predict. There is overfitting in the model. The importance of lncRNAs as predictive factors is lowered. Coefficients of duration of diabetes and patient age are low‐ranking	Canada
6	Transformer‐enhanced retinal vessel segmentation for diabetic retinopathy detection using attention mechanisms and multi‐scale fusion [86]	A novel segmentation model for retinal vascular delineation aimed at diagnosing diabetic retinopathy	DRIVE dataset. CHASEDB1 dataset. STARE dataset. HRF dataset. IOSTAR dataset. LES‐AV dataset	The study evaluates performance using metrics such as Mean IoU, Recall, and F1 score. F1 score is the harmonic mean of precision and recall. The proposed model achieves a F1 score of 0.7824	The proposed novel segmentation model improves feature extraction and representation, it is superior to the current segmentation approaches in terms of retinal vascular segmentation measurements, and the attention mechanisms are used to gain a better contextual perspective. Also needs more investigation into how the phases of design are integrated, and difficulties in representing local details and global dependencies continue to exist	Republic of Korea
7	Segmentation using the IC2T model and classification of diabetic retinopathy using the Rock Hyrax swarm‐based coordination attention mechanism [87]	The model uses a dual convolutional transformer architecture	Messidor‐2. DiaRetDB0. Kaggle DR 2015 competition (Dataset 1; 34,984 Fis). APTOS 2019 datasets (3662 Fis)	The H.M. model achieved mIoU of 0.86 and accuracy of 1.00. The HE model reached mIoU of 0.88 and recall of 0.88. The M.A. model had mIoU of 0.71 and accuracy of 0.71. The O.D. model obtained mIoU of 0.86 and accuracy of 0.86. The proposed technique achieved an average accuracy of 96%–98% on datasets	The model is highly accurate in DR detection (up to 98%), it uses a superior IC2T model to perform segmentation and classification, and it leverages local and global context to produce better results. It is based upon a complicated model architecture, which may expand the computation requirements, and cannot be tested and validated on arbitrary datasets	India
8	Improved detection accuracy of red lesions in retinal fundus images with a superlearning approach [88]	The model uses adaptive thresholding for red lesion segmentation. Ensemble‐based superlearning approach improves multiclass detection accuracy	Kaggle dataset. DiaRetDB1 database. Messidor database	The overall accuracy of red lesion segregation is 93.5%. Accuracy increases to 97.88% when addressing data imbalance	Super learning is quite effective in differentiating lesions and non‐lesions with a high accuracy of 93.5 and may increase to 97.88 with data processing. Indicators of performance can be compared to current literature‐based strategies, and lack of balance in the data can influence feature‐based forecasts	India
9	Multiclass adaptive boosting approach for diabetic retinopathy prediction using diabetic retinal images [89]	The model employs multiclass adaptive boosting for classification. The VGG16 pretrained model is used for feature learning	DR dataset. Kaggle dataset	The model achieved an accuracy rate of 91.6% with a kappa score of 0.883	A new multiclass adaptive boosting model is a better way of classifying data. It applies the fully trained VGG16 model in learning effective features and overfitting problems of CNN classification. Some challenges still exist in terms of limited CNNs performance in some situations	India
10	Reliable and energy‐efficient diabetic retinopathy screening using memristor‐based neural networks [90]	The model uses diverse image sources for training	EyePACS dataset. APTOS dataset	Key metrics include accuracy, F1 score, and energy consumption. Accuracy measures correctly classified retinal images as a percentage. F1 score reflects model’s prediction accuracy and minimizes false alarms. Energy consumption is crucial for deploying DR screening on edge devices	Various image sources were used to design a reliable DR classification model. It enhanced the balance of classes, increased postdeployment reliability, and deployed edge devices over energy efficient hardware design. Classifiers could perform poorly once deployed, and they do not have additional information regarding prediction results	Netherlands
11	Enhancing diabetic retinopathy detection through preprocessing and feature extraction with MGA‐CSG algorithm [91]	The model utilizes the MGA‐CSG approach for disease prediction. It combines CNN‐based feature extraction with GAN‐based augmentation	Fundus image dataset for diabetic retinopathy detection	Accuracy achieved was 98.7% in diabetic retinopathy detection. Precision rate recorded was 98.5%. Recall rate was 97.1%. Specificity rate reached 96.9%. F1 measure was 97.4%. The kappa coefficient was 93%. Mean square error (MSE) was 160.125	A robust DR classification framework was established utilizing varied image datasets. It augmented class equilibrium, fortified postdeployment dependability, and facilitated energy‐efficient hardware configurations for edge device integration. Classifiers may experience performance deterioration postdeployment and may lack additional insights regarding predictive results	India
12	Enhancing semisupervised contrastive learning through saliency map for diabetic retinopathy grading [92]	The model employs a semisupervised contrastive learning framework. It integrates an importance estimation map for enhanced focus	DDR dataset—Comprising 13,673 fundus images, with a focus on a five‐class DR grading task after excluding ungradable images. Messidor dataset—comprising 1200 fundus images, graded into four severity levels, with a binary classification strategy for DR grading. EyePACS dataset—mentioned as a publicly available DR grading dataset used for experiments	The performance metrics include accuracy and quadratic weighted kappa (kappa). SCL‐SEM outperforms state‐of‐the‐art methods on EyePACS and DDR datasets. The proposed method improves DR grading effectiveness through innovative technique	The research combines semisupervised and contrastive learning to enhance diabetic retinopathy (DR) grading precision. It successfully mitigates data limitations in medical imaging. The saliency map contributes positively to the model’s efficacy in identifying DR lesions, while the discourse on the methodology’s possible drawbacks remains minimal	China
13	Diabetic retinopathy detection using bilayered neural network classification model with resubstitution validation [93]	The study uses a bilayered neural network (BNN) model	Messidor, IDRiD, Kaggle EyePACS, APTOS	The model achieved 98.5% accuracy using resubstitution validation. The proposed method produced a ROC curve of 0.99. The system reached 98.25% specificity and 98% sensitivity. The highest accuracy recorded was 98.06% with 100% specificity	The system demonstrates a remarkable accuracy of 98.5% and a ROC curve value of 0.985 based on 6332 diabetic retinopathy images. The research underscores the critical role of timely intervention with appropriate DR treatment in averting blindness	Iraq
14	CMNet—cascaded context fusion and multiattention network for multiple lesion segmentation of diabetic retinopathy images [94]	The model proposed is a cascaded context fusion and multiattention network (CMNet)	IDRiD, DDR, and E‐Ophtha	The study used dice, IOU, HD, AUC_PR, and mAUC as metrics. AUC_PR indicates the area under the precision–recall curve mAUC represents the average AUC across different lesion types	The proposed CMNet demonstrates significant generalization across datasets. It proficiently segments multiple lesions with enhanced accuracy and robustness. However, the model has not been validated on retinal images of Black individuals and may be affected by background noise in certain scenarios	China
15	Computer‐aided diagnosis of diabetic retinopathy lesions based on knowledge distillation in fundus images [95]	The study employs a knowledge distillation‐based classification model for efficiency	Dataset with 1000 images belonging to 39 different categories, characterized by an imbalance and nonuniform distribution. Dataset containing 3592 images across eight different categories, maintaining a balance between them	Accuracy (ACC) and F1 score were used as performance metrics. ACC measures correct samples relative to total samples. F1 score accounts for recall and precision, especially in imbalanced datasets. Both metrics range from 0 to 1, with higher values indicating better performance	The DR‐CCTNet model attained a test accuracy of 90.17%. It proficiently classifies low‐resolution fundus images. The model markedly decreases training duration and employs an extensive dataset comprising 53,185 images. The model’s efficacy with real‐time data remains unexamined, and enhancements could enhance the quantity of raw images	Mexico
16	Classification of diabetic retinopathy algorithm based on a novel dual‐path multimodule model [96]	The proposed model is a dual‐path multimodule network for DR classification	APTOS2019 dataset (DR1). Messidor‐2 + EyePac dataset (DR2). Hospital dataset	The algorithm’s performance is evaluated using a confusion matrix. Metrics include accuracy, sensitivity, specificity, F measure, and kappa statistic. Higher values of these metrics indicate better model performance	The DP2M‐Net algorithm shows high accuracy in diabetic retinopathy classification. The algorithm is robust and generalizable for clinical applications. Future improvements are needed for model interpretability and transparency, and deep learning models are often perceived as black boxes	China
17	Application of nonmydriatic fundus photography‐assisted telemedicine in diabetic retinopathy screening [97]	The study evaluates NMFP‐assisted telemedicine for DR assessment. NMFP includes single‐field, double‐field, and seven‐field photography	Clinical data from 93 diabetic patients diagnosed at the First Affiliated Hospital of University of Science and Technology of China between June 2019 and June 2021. Diagnostic results from NMFP‐assisted telemedicine and fundus fluorescein angiography (FFA) for assessing diabetic retinopathy (DR)	Area under the ROC curve was 0.955 (*p* < 0.001). Specificity of NMFP‐assisted telemedicine was 100%. Sensitivity was recorded at 90.9%. The Youden index was 0.909. The kappa value for diagnostic consistency was 0.775. The kappa value for staging consistency was 0.689	NMFP‐assisted telemedicine shows high predictive accuracy for DR detection. Specificity of NMFP‐assisted telemedicine is 100%. Sensitivity of NMFP‐assisted telemedicine is 90.9%. Study enhances clinical accuracy in DR management. Small sample size limits the generalizability of findings and lacks cross‐sectional comparison with other DR screening methods	China
18	Artificial intelligence (AI)‐enhanced detection of diabetic retinopathy from fundus images [98]	AI models utilize deep learning for DR detection. Convolutional neural networks achieve high sensitivity and specificity. Multitask learning detects and grades DR severity simultaneously	APTOS dataset. EyePACS dataset	Deep DR achieved AUCs of 0.901–0.967 for various lesions. The model demonstrated high accuracy (98.0% and 98.4%) on APTOS and EyePACS. AUCs for DR grading ranged from 0.943 to 0.972	AI systems show expert‐level accuracy in detecting diabetic retinopathy. The challenges include generalizability across diverse populations and data quality. Ethical considerations and healthcare disparities are significant weaknesses, and integration into clinical workflows presents technical and organizational challenges	Pakistan
19	A computer‐aided diagnostic system to identify diabetic retinopathy, utilizing a modified compact convolutional transformer and low‐resolution images to reduce computation time [99]	The proposed DR‐CCTNet model	APTOS Messidor‐2 IDRiD DDR Kaggle diabetic retinopathy	The proposed model achieved a test accuracy of 90.17%A	The DR‐CCTNet model outperformed conventional models in accuracy. The model is robust with low‐pixel images. Future research could enhance performance with more raw images, and minor shortcomings exist in the proposed model	Bangladesh
20	A meta‐learning approach for diabetic retinopathy severity grading [100]	The proposed methodology includes preprocessing and feature extraction processes. A multipath convolutional neural network technique is utilized	APTOS dataset. Kaggle dataset	The ML‐ERNN achieved 98% precision on the APTOS dataset. The ML‐ERNN achieved 98% precision on the Kaggle dataset. The f‐measure for ML‐ERNN was 98.98% on the APTOS dataset. The f‐measure for ML‐ERNN was 98.98% on the Kaggle dataset	The model utilizes advanced meta‐learning techniques for improved accuracy. It employs weighted stacking‐based ensemble classifiers for better performance. Focuses on early detection of diabetic retinopathy, and high time complexity may affect classifier performance	India
